# Non-thermal plasma activated water is an effective nitrogen fertilizer alternative for *Arabidopsis thaliana*

**DOI:** 10.1371/journal.pone.0327091

**Published:** 2025-09-08

**Authors:** Jonathan Kizer, Conner Robinson, Ta’Kia Lucas, Steven Shannon, Ricardo Hernández, Katharina Stapelmann, Marcela Rojas-Pierce

**Affiliations:** 1 Department of Plant and Microbial Biology, North Carolina State University, Raleigh, North Carolina, United States of America; 2 Department of Nuclear Engineering, North Carolina State University, Raleigh, North Carolina, United States of America; 3 Department of Horticultural Sciences, North Carolina State University, Raleigh, North Carolina, United States of America; Shahrekord University, IRAN, ISLAMIC REPUBLIC OF

## Abstract

Nitrogen (N) fixation with non-thermal plasmas has been proposed as a sustainable alternative to meet growing N fertilizer demands for agriculture. This technology generates Plasma Activated Water (PAW) with a range of chemical compositions, including different concentrations of nitrate (NO₃⁻) and hydrogen peroxide (H_2_O_2_), among other compounds. Potential use of PAW as an effective crop fertilizer necessitates a robust understanding of the underlying biology of the plant, which is not yet available. The lack of a unified standard in PAW production and the varying chemical make-up that results from different devices and protocols hampers comparative studies and adoption of this technology. The objective of this study was to compare the efficacy of two PAW solutions with differing concentrations of H_2_O_2_ produced from a Radio Frequency (RF) glow discharge plasma source. The effect of these solutions on plant growth, ROS accumulation, gene expression and heat stress response were compared to N-equivalent controls in the model plant Arabidopsis to assess their potential as an alternative N fertilizer. While PAW solutions lacking detectable H_2_O_2_ enhanced seedling growth, those containing approximately 0.3 µM of H_2_O_2_ did not. ROS accumulation in root tissues was similar between PAW and chemically equivalent solutions, suggesting H_2_O_2_ is the primary ROS present in the PAW at the time of treatment. Gene expression studies showed induction of genes involved in N uptake and assimilation in PAW-treated seedlings. Pre-treatment of seedlings with PAW solutions containing H_2_O_2_ improved root growth under heat stress which indicates that this treatment may induce plant stress response pathways. Finally, mature plants showed similar growth when fertilized with PAW lacking H_2_O_2_ or NO_3_^-^ control regimes for over 5 weeks indicating equivalency in chemical composition, plant nutrient uptake and utilization. Overall, these results demonstrate that PAW is an effective alternative to NO_3_^-^ fertilizers for plant cultivation but the levels of H_2_O_2_ need to be carefully controlled.

## Introduction

The demand for nitrogen (N) fertilizer is concomitant to the rising global demands for food [1]. For the past 50 years, synthetic fertilizers have made up the largest portion of N fertilizers utilized in global agriculture [1]. The most used method to fix N for fertilizers is the Haber-Bosch process which is energy intensive, reliant on fossil-fuels, and contributes 2.9 tons of atmospheric CO_2_ globally per year [2]. Alternative N-fixation strategies such as Non-thermal plasmas (NTP) are being investigated to enhance the sustainability of agricultural practices, while meeting growing fertilizer demands [3]. NTP can fix gaseous atmospheric N_2_ into water, creating plant-available N forms. Additionally, NTP treatment of water produces reactive oxygen and nitrogen species (RONS). The specific parameters of the treatment process influence the resulting chemistry and concentration of these RONS [4–6], which are also relevant to plant health. This resulting solution is referred to as Plasma-Activated Water (PAW) or Plasma-Treated Water (PTW).

Plant health is dependent on the availability of nutrients in their environment. Chief among these nutrients are the macronutrients nitrogen (N), phosphorus (P), and potassium (K), with N being vital for plant productivity [7]. Plants preferentially take up NO_3_^−^ and NH_4_^+^ from the soil, with NO_3_^−^ being a major source for N for many plants [8]. Roots primarily take up NO_3_^−^ via high- and low-affinity transporters located in the plasma membrane, including those from the NRT1 and NRT2 protein families [9]. Once in the cytosol, NO_3_^−^ is first reduced to NO_2_^−^ by the enzyme nitrate reductase, and then transported to the chloroplast to be further reduced to NH_4_^+^ by nitrite reductase. This NH_4_^+^ can then be incorporated into organic N compounds such as amino acids [9]. NH_4_^+^ can also be taken up directly from the soil via high- and low-affinity transporters. Ammonia Transporter (AMT) family transporter proteins are primarily responsible for NH_4_^+^ uptake in the root [10]. The expression of AMT transporters, is highly regulated and controlled by factors such as light, external pH, and internal NH_4_^+^ levels [11].

Nitrogen uptake and assimilation are regulated by N availability in the immediate soil environment. NO_3_^−^ functions as a signal molecule that ultimately controls the response to N availability. The perception of this signal leads to downstream responses involving transcriptional, post-transcriptional, and post-translational control [12]. Some members of the NRT1 and NRT2 family of membrane transporters have additional functions as NO_3_^-^ receptors, with NRT1.1 likely playing this role in root cell plasma membranes [13,14].

NO_3_^-^ influences the development and morphology of both belowground and aboveground organs [13]. Low nitrate concentrations (< 1 mM in *Arabidopsis thaliana*), promote elongation in both primary and secondary roots, and this is described as a “foraging” response. This response increases the root surface area and enhances uptake of NO_3_^-^ from surrounding soil. In contrast, high NO_3_^-^ conditions (> 10 mM in *A. thaliana*) reduces root elongation, and inhibits lateral roots elongation at the seedling stage [12]. These morphological responses are integrated alongside phytohormone responses. N-driven lateral root initiation is regulated by the crosstalk between NO_3_^-^ and auxin pathways [14]. Moreover, the NO_3_^-^ transceptor NRT1.1 likely has auxin transport capability, in which it basipetally transports auxin from the lateral root primordia, preventing its accumulation and the subsequent elongation of lateral roots in response to ample NO_3_^-^ [13,15]. Overall, the response to NO_3_^-^ is highly regulated and dependent on the availability of the nutrient in the root environment.

Reactive oxygen species (ROS) are naturally produced in chloroplast and mitochondria in photosynthetic tissues and mainly mitochondria in non-photosynthetic tissues. In plant cells, ROS are produced as byproducts of natural inefficiencies of the electron transport chain. Peroxisomes, glyoxysomes, and the apoplast are also sites of ROS production with the latter occurring via cell wall peroxidases and plasma membrane NADPH oxidases. While prevalent in the cell, excessive ROS can be detrimental to plant growth by distorting redox homeostasis and potentially damaging proteins, cellular membranes, and nucleic acids [16]. Plant cells maintain several scavenging mechanisms to curtail excessive damage from excessive ROS, including enzymes that catalyze the breakdown of the reactive species to more stable species. These include Superoxide Dismutases (SOD) which catalyze the conversion of superoxide (O_2_^-^) to H_2_O_2_ [17,18] and Catalases (CAT) which catalyzes the conversion of H_2_O_2_ into H_2_O [19–21]. Additionally, there is ample evidence that ROS are required in biological systems [16,22]. For example, hydrogen peroxide (H_2_O_2_) and superoxide (O_2_^-^) modulate plant growth, development [23–25] and stress responses [26].

Both reactive oxygen and nitrogen species (RONS) can function as signaling molecules and lead to acclimation to varying abiotic stresses [23,27,28]. Abiotic stressors may result in a build-up of RONS beyond the scavenging ability of antioxidants, eliciting responses at the cellular and whole-plant levels [29,30]. Incidentally, introduction to RONS prior to a stress event can induce tolerance to that stress including nutrient and water deficit, a response known as “priming” [31–33]. Altogether, these complex responses to exogenous and endogenous RONS may be leveraged to improve growing practices in stressful environments [34,35].

N fixation via non-thermal plasma represents an alternative to supplement growing global demands for NO_3_^-^ [3,36–40]. Studies of the effects of PAW on varying crop species have begun to describe the potential of plasma agriculture [37,41–44]. Several studies have shown increased growth in PAW-treated crops, with cereal crops notably showing significant height differences compared to untreated controls [37,43]. However, while several of these studies hypothesized that plant growth differences are due to the ROS present in PAW, few studies have delved deeper into the underlying biology. Studies in model plant systems do exist with some focusing more on direct-plasma treatment of seedlings [45,46]. Others in the field have begun specifically investigating the underlying effects of PAW-treatment on the plant response [47,48] and how these responses can bolster the efficacy of PAW as a supplemental source of N. However, the field of plasma agriculture has no unified standard for quantifying PAW’s impacts on plants, making it difficult to determine the applicability of these effects across other systems and environments.

Prior studies have not addressed the plant response to PAW in detail, especially by comparing to equivalent NO_3_^-^ fertilizer regimes and testing the effects of different levels of H_2_O_2_. Moreover, it is unclear whether PAW treatments alter signaling pathways typically regulated by NO_3_^-^_._ This study compared the Arabidopsis response to multiple PAW chemistries with equivalent fertilizer regimes using traditional sources of N. Overall, similar growth was detected in PAW-treated plants when compared to NO_3_^-^ controls, but negative effects were detected for H_2_O_2_ in some PAW chemistries_._ Fluorescence microscopy and transcriptome analyses demonstrated that PAW treatment did not alter the ROS or hormone response signaling pathways. Interestingly, certain PAWs appear to function in “priming” as enhanced tolerance to heat stress was observed in seedlings pre-treated with H_2_O_2_-containing PAW. Our studies underscore the potential of PAW as an alternative to NO_3_^-^ fertilizers in plants but H_2_O_2_ levels must be measured and evaluated in specific plant growth systems.

## Materials and methods

### PAW production

Plasma water treatments were conducted using an atmospheric radio frequency (RF) glow discharge plasma, as described in [49,50]. This plasma device was used to surface treat bulk diH_2_O volumes to produce the PAW used in these experiments. An AE OVAtion 35162 RF Generator is used to power the device, and the corresponding software is used to control the delivered power. For all treatments, the delivered power was kept constant at 250 W. Air was flowed down the coaxial electrode, toward the water surface at a rate of ≤ 1 slm (this was controlled with an analog gauge which did not read below this value and would vary depending on the background pressure from the buildings compressed air supply). It is recommended, for reproducibility, that flow rates be more accurately controlled/monitored, which could be obtained by using a mass flow controller. Treatment parameters varied depending on the desired chemistry, namely the inclusion of H_2_O_2_. To optimize the production of NO_3_^-^, a large external volume of water (≥ 2 L, diH_2_O, pH ≈ 5.2, EC ≈ 2μS) was circulated through the plasma chamber – which was kept open to improve ventilation – and the distance between the water surface and electrode was set to minimize reflected power (20–40 W reflected at 1.5 cm). Under these conditions, the plasma was consistently able to produce aqueous NO_3_^-^ at a rate of 2 mg/min and treatment times were adjusted accordingly to achieve the desired concentration for the target volume. For H_2_O_2_ production, the plasma chamber was sealed with a smaller, stagnant water volume (450 mL), and the gap distance was reduced to roughly 0.75 cm (100–200 W reflected). This would result in significant evaporation, lowering the water surface and increasing the gap distance as the treatment progressed. The reflected power corresponded closely with the gap distance (when other parameters are kept constant) and was used to determine when the gap distance had changed significantly. The water volume was placed upon a lab jack within the chamber, and when the reflected power dropped below the desired range, the jack would be raised by hand while the treatment was ongoing. To improve reproducibility in the future, the authors recommend employing a system to ensure the water level (gap distance) remain constant autonomously, such as with a ballasted pumping system. These treatments lasted 45 minutes, with 250 ml of diH_2_O remaining by the end. This PAW typically contained 50 mg/L of H_2_O_2_ and 50–100 mg/L of nitrate NO_3_^-^; however, this was far less consistent than the NO_3_^-^ focused treatments. Treatments were repeated with fresh diH_2_O until the amount of aqueous H_2_O_2_ necessary to achieve the desired concentration for the target volume was produced. The NO_3_^-^ only and NO_3_^-^ + H_2_O_2_ PAW were initially kept separate, their chemical composition measured colorimetrically and then were combined with one another and/or untreated diH_2_O to obtain the final desired PAW volumes and chemistries. The final PAWs were also tested colorimetrically to confirm their composition. NO_3_^-^, NO_2_^-^, H_2_O_2_, and NH_4_^+^ levels were tested for each step in triplicate using the commercially available Supelco test kits: 1.09713, 1.14776, 1.18789 and 1.14752, respectively. Absorbance values were obtained using an UV-VIS-NIR light source (Ocean Optics DH-2000-BAL) in conjunction with a spectrometer (Ocean Optics QE65 Pro) and a cuvette holder (Ocean Optics CUV-UV). These absorbances were converted to concentrations using stock solution based standard curves prepared in advance. PAW was neutralized with 1M KOH solution to increase the pH to 5.7, a plant-viable pH. Neutralization maintained stability of the solution for storage. PAW was stored in the dark at room temperature for up to 2 weeks before use. While PAW stability was not measured at 2 weeks of storage, other studies of PAW chemical longevity [51–54]suggest minimal changes to PAW chemistry.

Plant material and growth conditions

*Arabidopsis thaliana* ecotype Columbia 0 (Col-0) was used for all experiments. Arabidopsis lines expressing the hormone response markers DR5::GFP [55] or TCSn::GFP [56] were obtained from the Arabidopsis Biological Resource Center (ABRC). The EBS:Ypet marker line was previously described [57]. Seeds were surface sterilized with 95% ethanol followed by a solution containing 20% commercial bleach and 0.1% Tween 20 (VWR, MFCD00165986). Seeds were rinsed 2–3 times with sterile diH_2_O and then stored at 4 °C for 4 days in the dark. Seeds were then plated onto Arabidopsis Growth Media (AGM) containing 0.5 x MS with MES (Murashige & Skoog, 1962, RPI, M70300), 1% sucrose and 4g/L Gelrite (RPI, G35020). Plates were incubated vertically in a growth chamber with 120 µmol/m^2^/s of PPFD at 22 °C with a 16 h/8 h day/night cycle to promote germination. Plants potted in soil were grown in growing benches with LED grow lights under similar controlled conditions as plated plants. PPFD was provided at 130 µmol/m^2^/s at plant height. Plants grown on soil were rotated to new positions regularly on grow benches to reduce impact of uncontrolled environmental effects.

### PAW and NO_3_^-^ control treatments

AGM with PAW or NO_3_^-^ for seedling treatments was prepared as follows: 1 X (4.3 g/L) MS media without nitrogen (MS-N, Bio-World, 30630200) was adjusted to pH 5.7, supplemented with 8 g/L Gelrite and 2% sucrose and then sterilized by autoclaving (2X AGM-N). Control solutions were prepared in diH_2_O using potassium nitrate (KNO_3_^-^) (Caisson Labs, P012) and 30% (w/w) H_2_O_2_ solution (Sigma-Aldrich, H1009) at the same concentrations of pre-mixed PAW (4.8 mM or 3.5 mM NO_3_^-^ with or without 0.3 mM H_2_O_2_). All treatment solutions including PAW were gently brought up to 60 °C in a water bath and then filter sterilized using a 0.45 µm pore filter inside a laminar flow hood. The 2X AGM-N media was melted in a microwave and cooled to 60 °C just before mixing. Treatment solutions were then mixed with an equal volume of 2X AGM-N media in a pre-heated bottle and the mixture was rapidly poured into square petri dishes in the laminar flow hood.

PAW treatments at the seedling stage were performed by transferring 4-day old seedlings from AGM media to AGM containing PAW or control solutions and incubated for 5–7 days or as indicated. PAW treatments in soil were achieved by irrigation of nutrient solutions as follows. A substrate mixture devoid of N was used to avoid any unspecified fertilizer normally present in commercial soil mixes. The soil mixture contained 45% peat moss (Premier Peat Moss), 35% vermiculite (Sta-Green Vermiculite), and 20% perlite (Aero Soil Perlite) (all measured by volume). Soil components were not amended with nutrients as per the manufacturer’s label. Pulverized limestone (Gardenlime) was added to adjust pH to ~6.0. The soil was moistened with diH_2_O and distributed into 2-inch insert pots (T.O. Plastics, 2401 Standard). Three-day old seedlings previously germinated on sterile AGM media were transferred to each pot of moistened soil. Seedlings were thinned to 1 per pot 3 days after transfer. All seedlings were watered with 50 ml diH_2_O per pot twice weekly to keep the plants hydrated. 50 ml per pot of 0.25x Hoagland Solution without N (Bio-World, 30630038) was given to all seedlings biweekly by top irrigation. Specific treatments were provided by top irrigation with 50 ml per pot of either PAW, equivalent NO_3_^-^ control solution, or Low N (0.5 mM NO_3_^-^) control solution once per week.

### Plant growth measurement

Seedling plates were scanned using a desktop scanner every 2 days and the primary and lateral root lengths were measured using ImageJ software version Fiji [58]. Total root length was calculated by summing the primary and lateral root lengths. Lateral root density was calculated by dividing the number of lateral roots of a given individual by the primary root length.

After 5 weeks of treatment, soil-grown plants were imaged top-down with a 12 MP digital camera positioned 18 cm above the soil. These images were used to calculate rosette area using Fiji. After imaging, soil-grown plants were harvested, and shoots were separated from roots. Aboveground tissue fresh biomass was obtained immediately. Then shoots and cleaned root samples were dried at 175°C for 12 hours to determine dry biomass.

### Heat stress experiments

Four-day old seedlings, grown as described above, were transferred onto PAW or control treatment plates and incubated for 1 day at 22°C. Heat stress was achieved by transferring half of the plates to an incubator set at constant temperature of 30°C while keeping identical PPFD and day/night lighting cycles. The remaining plates were kept at 22°C as controls. After 4 days, all plates were scanned with a desktop scanner, and their root lengths were measured using ImageJ. The experiments were replicated 3 times with similar results.

### Fluorescent probe staining, imaging, and quantification

Four-day old seedlings were transferred onto AGM media containing PAW, NO_3_^-^ or NO_3_^-^ combined with H_2_O_2_ (0.15mM or 10mM) and stained 2 minutes, 2 hours or 2 days later with 2’,7’ Dichlorodihydrofluorescin Diacetate (H_2_DCFDA, Sigma-Aldrich, 287810) or Peroxy Orange 1 (PO1, Tocris Bioscience, 49-441-0). H_2_DCFDA and PO1 were dissolved in DMSO as 10 mM stocks. Dyes were diluted to 10 μM (H_2_DCFDA) or 50 μM (PO1) working solutions in 0.5 x AGM-N liquid media and seedlings were stained in the dark for 10 min (H_2_DCFDA) or 30 min (PO1) before one rinse with water and imaging in the microscope. Stained seedlings were imaged on a Zeiss LSM 980 confocal microscope using a 20x objective (Zeiss Plan-Apochromat/ 0.8 N.A.). H_2_DCFDA was excited with a 488 nm laser at 0.25% power and emission was collected at 509–550 nm. PO1 was excited with a 488 nm laser at 0.1% power and emission was collected at 544–695 nm.

### RNA sequence analysis

Three-day old Col-0 Arabidopsis seedlings were transferred onto AGM containing PAW4 or NO_3_^-^ and grown for additional 8 days. The shoot and root tissue of these seedlings were excised, and excess gel media was removed immediately by gentle blotting on low-lint tissue paper. Samples were pooled into four replicates of approximately 20 Arabidopsis seedlings each and placed in 5 volumes of RNAlater stabilization solution (Thermo Fisher Scientific, AM7020) based on tissue mass as specified by the manufacturer. Seedlings spent less than 10 seconds from harvesting to transfer to RNAlater. RNA was then extracted from the tissue using the RNeasy plant mini kit (Qiagen, 74904). Quality of extracted RNA was tested utilizing an Agilent 4200 Tapestation. cDNA library preparation was performed for samples with passing RIN scores using the NEB NEBNext Ultra II Direction RNA kit (polyA enriched) (NEB, E7760S). Next-Generation Sequencing was conducted using an Illumina NovaSeq 6000 with 150-base paired-end reads. The resulting sequencing data was processed and analyzed utilizing the Qiagen CLC Genomics workbench. Expression data for root and shoot data was analyzed for replicates treated with PAW4 against replicates treated with the NO_3_^-^ control. Root and shoot tissues were analyzed separately. Gene ontologies were investigated and plotted utilizing the ShinyGO (version 0.82) bioinformatics tool with a focus on biological processes [59].

### Statistical analysis

Statistical analysis was performed in this study utilizing the R programming language (R-Project). For root length comparisons, a one-way ANOVA with significance at a 0.05 alpha was performed to determine significant differences in length across multiple treatments. A Tukey HSD test was performed as a post-hoc analysis to determine specific relationships between treatments. Tukey HSD was chosen for the test’s effectiveness when performing pairwise comparisons and ability to reduce false positive errors [60]. For experiments comparing the effects of treatment and elevated temperature on root length, a two-way ANOVA was conducted. Comparisons were made across media treatments and temperatures. Tukey HSD was conducted as a pos-hoc analysis.

Analysis of tissue biomass, plant heights, and rosette areas was performed with a Student’s T-test. All data was assumed normal, and significance was denoted at a 0.05 alpha.

## Results

### Optimal PAW treatments promote total root elongation in seedlings outside the effects of NO_3_^-^ and H_2_O_2_

To evaluate how different PAW chemistries affect plant growth, a Radio Frequency (RF) glow discharge plasma source [49,50]. was used to produce PAW with differing concentrations of NO_3_^-^ or H_2_O_2_ (Table 1). This allowed for pairwise comparisons. NO_2_^-^ and NH_4_^+^ levels were very low in all PAWs and ranged between 2–4 mg/L immediately after plasma treatment (S1 Fig in S1 File). After incorporation into gel media, PAW treatments contained either 1.75 mM (110 ppm, PAW1 and PAW2) or 2.4 mM (150 ppm, PAW4 and PAW5) of NO_3_^-^, concentrations known to be sufficient for Arabidopsis growth [61]. Within each pair, PAWs differed by the presence of 0.15 mM H_2_O_2,_ enabling a systematic assessment of this ROS. The RF glow discharge plasma source was suitable for producing 2 mg/min NO_3_^-^ in deionized H_2_O and thus was feasible for plant experiments.

**Table 1:** Concentrations of NO_3_^-^ and H_2_O_2_ in treatment solutions.

ID	NO_3_^-^ (mM)	H_2_O_2_ (mM)
diH_2_O	0	0
Low N	0.25	0
NO_3_^-^	2.4	0
H_2_O_2_ + NO_3_^-^	2.4	0.15
PAW1	1.75	0
PAW2	1.75	0.15
PAW4	2.4	0
PAW5	2.4	0.15

Expected concentration of NO_3_^-^ and H_2_O_2_ from each PAW or control solution after incorporation into gel media. The solutions consist of the Low N, NO_3_^-^, and H_2_O_2_ + NO_3_^-^ treatments were mixed from NO_3_^-^ salts and diluted H_2_O_2_ to match the concentrations of the PAW treatments.

**Fig 1:**
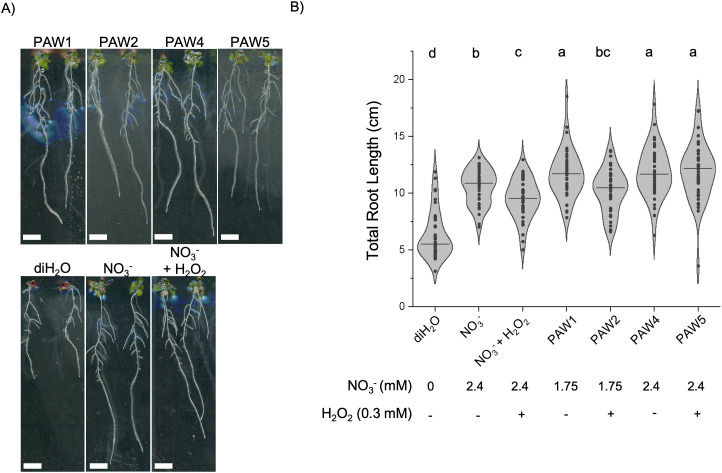
Optimal PAW treatment promotes total root elongation of Arabidopsis seedlings. A) Three-day old Arabidopsis seedlings were transferred to N-free media supplemented with PAW1, PAW2, PAW4 or PAW5. As controls, same age seedlings were transferred to N-free media without N supplementation (diH_2_O) or supplemented with 2.4mM NO_3_^-^ or 2.4mM NO_3_^-^ and 0.15mM H_2_O_2_. Seedlings were imaged after 5 days using a scanner. B) The root length of primary and secondary roots combined was measured from seedlings treated as in A). Final levels of NO_3_^-^ or H_2_O_2_ in the media are indicated under each treatment. A one-way ANOVA with Tukey’s multiple comparison test was performed. N = 40 seedlings. Significant differences are indicated using a compact letter display.

PAW treatments were applied to 4-day-old Arabidopsis (Col-0) seedlings to circumvent well-documented effects of PAW on seed germination [45,62–64]. Seedlings were transferred to N-free media supplemented with PAW, diH_2_O (negative control), 1.75 mM NO_3_^-^ or 1.75 mM NO_3_^-^ combined with 0.15 mM H_2_O_2_. Seedling growth was measured after 5 days of treatment (9 days after germination). As expected, plants treated with NO_3_^-^ controls or any PAW solution showed a significant increase in root length compared to diH_2_O controls (Fig 1A, B). PAW1 and PAW4 treatments, which contain 1.75 and 2.4 mM NO_3_^-^, respectively, but no measurable H_2_O_2_, showed ~11% increase in total root length compared to the NO_3_^-^ control. Interestingly, PAW2 treatment resulted in a 14% decrease in total root length on average when compared to PAW1, suggesting that H_2_O_2_ in this PAW may dampen root elongation. This effect was also evident in the shorter total and primary roots of the NO_3_^-^ plus H_2_O_2_ controls when compared to NO_3_^-^ alone. PAW5 treatments resulted in similar total root length as the PAW4-treated plants. Measurements of primary root length showed similar results where PAW2 and PAW5 treatments, which contain measurable NO_3_^-^ and H_2_O_2_, resulted in shorter primary roots compared to their counterparts without H_2_O_2_, PAW1 and 4, respectively (S2A Fig in S1 File). Therefore, PAW1, 4, and 5 showed increased total root growth compared to NO_3_^-^ and NO_3_^-^ plus H_2_O_2_ despite PAW5 having a reduced primary root growth (Fig 1B). To determine whether differences in total root length were due to secondary root initiation or elongation, lateral root density was measured in PAW-treated plants. PAW-treated plants showed similar or reduced lateral root density compared to both the NO_3_^-^ and NO_3_^-^ plus H_2_O_2_ controls. Given that seedlings treated with PAW1, PAW4, or PAW5 have longer roots without significant increases in lateral root density, the observed increases in root lengths appear to be primarily due to elongation rather than increased lateral root initiation (S2B Fig in S1 File). PAW4 and PAW5 were selected as the optimal PAW chemistries for further experiments due to the increased elongation seen in treated seedlings.

### ROS present in PAW inhibit root growth in arabidopsis seedlings

Previous research suggested that RONS in PAW solutions enhance plant growth [39,65]. However, both PAW2 and PAW5, which contain H_2_O_2_, showed shorter primary roots compared to their counterparts without H_2_O_2_. Moreover, the NO_3_^-^ content in PAW alone does not explain the differing root growth responses, as PAWs with different levels of H_2_O_2_ showed different levels of root growth. RONS are still a major component of PAW [63,66–68], with H_2_O_2_ being the most concentrated and stable RONS present in the PAW2 and PAW5.

**Fig 2:**
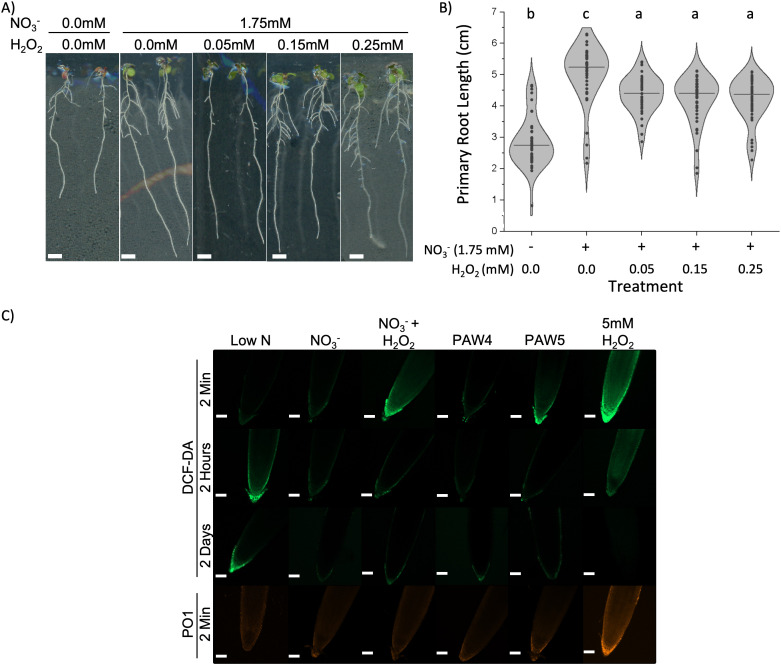
H_2_O_2_ was detrimental to root length. A) Three-day old Arabidopsis seedlings were transferred to N-free media supplemented with 0 (diH_2_O) or 2.4mM NO_3_^-^ and varying levels (0-0.25 mM) of H_2_O_2._ Plants were incubated for 5 days and imaged with a scanner. B) The primary root length was measured from images of seedlings treated as in A). The concentration of NO_3_^-^ or H_2_O_2_ in the media are indicated under each treatment. Data shows results of one-way ANOVA with Tukey’s multiple comparison test. N = 40 seedlings. Significant differences are indicated with compact letter display. C) Three-day old seedlings were incubated in N-free media supplemented with 0.25 mM NO_3_^-^ (Low N), 2.4 mM NO_3_^-^ (NO_3_^-^) with or without 0.15 mM H_2_O_2_, PAW4, PAW5, or 2.4 mM NO_3_^-^ with 5 mM H_2_O_2_ for 2 minutes, 2 hours, or 2 days and then stained with 2′-7′ dichlorodihydrofluorescein diacetate (DCF-DA) or Peroxy Orange 1 (PO1). Root tips were imaged by confocal microscopy (scale = 100 µm).

To further investigate the role of H_2_O_2_ on the plant response to PAW, root growth was measured in Arabidopsis seedlings (Col-0) treated with 1.75 mM NO_3_^-^ and varying concentrations of H_2_O_2_. Seedlings treated with up to 0.25 mM H_2_O_2_ appeared healthy with limited visual stress symptoms (Fig 2A), yet their primary roots were, on average, 18% shorter compared to seedlings treated with the NO_3_^-^ without H_2_O_2_ control. Even treatments as low as 0.05 mM H_2_O_2_ resulted in a 14% reduction in primary root length when compared to the NO_3_^-^ without H_2_O_2_ (Fig 2B). Ultimately, H_2_O_2_ in NO_3_^-^containing solutions resulted in inhibition of primary root growth.

Although H_2_O_2_ in PAW5 inhibited primary root elongation, other ROS potentially present in PAW could contribute to the elongated root response to PAW4. Cells in the root tip and surrounding tissue control root elongation by regulating cell division and cell elongation rates, which is in part regulated by ROS [22,26]. Therefore, it was important to investigate whether short PAW treatments elicited the accumulation of different ROS in root tips, which was accomplished with the fluorescent dyes 2′-7′ dichlorodihydrofluorescein diacetate (H_2_DCFDA), a general intracellular ROS probe [69,70], and Peroxy Orange 1 (PO1), a specific probe for intracellular H_2_O_2_ [70,71]. Seedlings transferred to N deficient media showed ROS accumulation in Arabidopsis root tips after 2 hours, as detected by H_2_DCFDA staining (Fig 2C) and consistent other studies [72,73]. No apparent changes in H_2_DCFDA fluorescence were detected up to 2 days after transfer of seedlings to N-replete media (NO_3_^-^ control) (Fig 2C). In contrast, seedlings transferred to media containing NO_3_^-^ plus H_2_O_2_ showed increased H_2_DCFDA fluorescence along the root cap and root tip epidermis, but only at the 2 minutes time point (Fig 2C). At later time points, reduced fluorescence was found across all treatments except the Low Nitrogen (Low N) control. The activity of scavenging enzymes, including catalases [74,75], may explain the reduction in H_2_DCFDA fluorescence at later times points. Roots treated with PAW4-containing media exhibited a similar pattern and intensity of H_2_DCFDA fluorescence compared to the NO_3_^-^ control at all time points (Fig 2C), suggesting the absence of other unaccounted ROS components. Conversely, PAW5-treated seedlings showed similar staining as the NO_3_^-^ + H_2_O_2_ control with stronger H_2_DCFDA after 2-minutes of seedling transfer but reduced fluorescence in later timepoints. This increase in H_2_DCFDA fluorescence 2 minutes after treatment with PAW5 or the NO_3_^-^ plus H_2_O_2_ control may be attributed to endogenous ROS production in response to the elevated H_2_O_2_ in the media, akin to a stress response [32,69,76,77]. A high (5 mM) H_2_O_2_ + NO_3_^-^ treatment was used to confirm successful probe incubation and resulted in high levels of H_2_DCFDA fluorescence throughout multiple cell layers of the root after 2 minutes. H_2_DCFDA staining also decreased in these seedlings after 2 hours or 2 days of treatment. No differences in Peroxy Orange 1 (PO1) fluorescence intensity were detected in seedlings treated with PAW4, PAW5 or the corresponding NO_3_^-^ controls with or without H_2_O_2_. PO1 staining was only detected in seedlings exposed to the 5 mM H_2_O_2_ control. This result suggests that the accumulation of endogenous H_2_O_2_ is not the primary ROS produced under PAW5 and the NO_3_^-^ plus H_2_O_2_ treatments. It is important to note that as a boronate-based probe, PO1 can react slower than H_2_DCFDA, which might make short-term ROS accumulation appear weaker as the probe needs more time to respond [70,78].

### PAW5 pre-treatment confers some protection to plants under heat response

**Fig 3:**
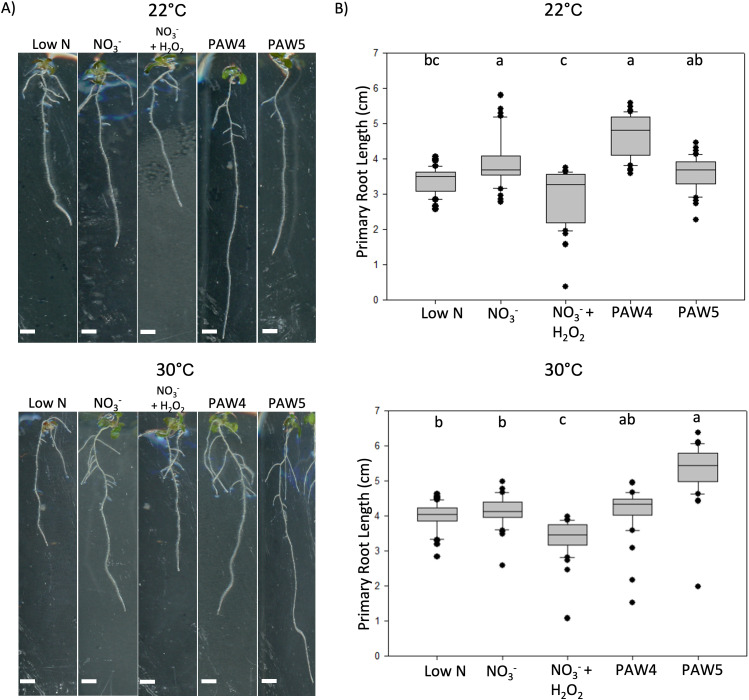
PAW5 confers resistance to heat stress. A) Three-day old Arabidopsis seedlings were transferred to N-free media containing 0.25 mM NO_3_^-^ (Low N), 2.4 mM NO_3_^-^ (NO_3_^-^), 2.4 mM NO_3_^-^ and 0.15 mM H_2_O_2_ (NO_3_^- ^+H_2_O_2_), PAW4 or PAW5. Plants were incubated for 1 day at 22°C to expose them to each treatment. After that, plates were either kept at 22°C (top) or incubated at 30°C (bottom) for 5 days to expose them to high temperature. Plates were imaged on a flat scanner. B) Primary root length was measured from seedlings at the end of the 22°C (top) or 30°C (bottom) incubation as described in A). Data represented as a boxplot showing the distribution of root lengths across all treatments. A one-way ANOVA with Tukey’s multiple comparison test was performed. Significant differences are indicated with compact letter display. N = 40 seedlings per treatment.

H_2_O_2_ has been linked to priming in plants, which can confer resistance to abiotic stressors [31–33,76]. The differing concentrations of H_2_O_2_ in PAW solutions allowed us to determine if PAW could provide a priming effect to protect Arabidopsis against elevated temperatures. Three-day old seedlings (Col-0) were transferred to PAW or NO_3_^-^ control media and incubated at 22°C for 1 day before moving them to 22°C or 30°C for an additional 4 days. The 22°C control set of seedlings exhibited root growth consistent with prior experiments, where PAW5-treated seedlings showed similar or reduced primary root length compared to both PAW4 and the NO_3_^-^ control (Fig 3A, B). Seedlings treated with high or low NO_3_^-^ showed similar root lengths at 30°C when compared within this temperature group (Fig 3B), suggesting that N availability did not impact the response to elevated temperatures. PAW4-treated seedlings showed similar root growth compared to NO_3_^-^ controls, indicating that PAW4 does not protect plants against heat stress (Fig 3B). Plants undergoing PAW5 treatment, which contains 0.15 mM of H_2_O_2_, showed a 46% increase in primary root length compared to the NO_3_^-^ plus H_2_O_2_ control at the end of the elevated temperature period (Fig 3B). PAW5-treated seedlings also had 27% longer primary roots compared to PAW4-treated seedlings after 4 days of heat stress (Fig 3B), which is the opposite to the effects observed at 22°C (S2A Fig in S1 File). This suggests that H_2_O_2_ in PAW5 elicited beneficial response to elevated temperatures compared to other treatments in that group. However, this cannot be fully explained by the H_2_O_2_ content in PAW5, as the NO_3_^-^ plus H_2_O_2_ control were not sufficient to cause the same improved growth under elevated temperatures.

### PAW treatment in seedlings shows no detectable change in hormone accumulation in root tissue

**Fig 4:**
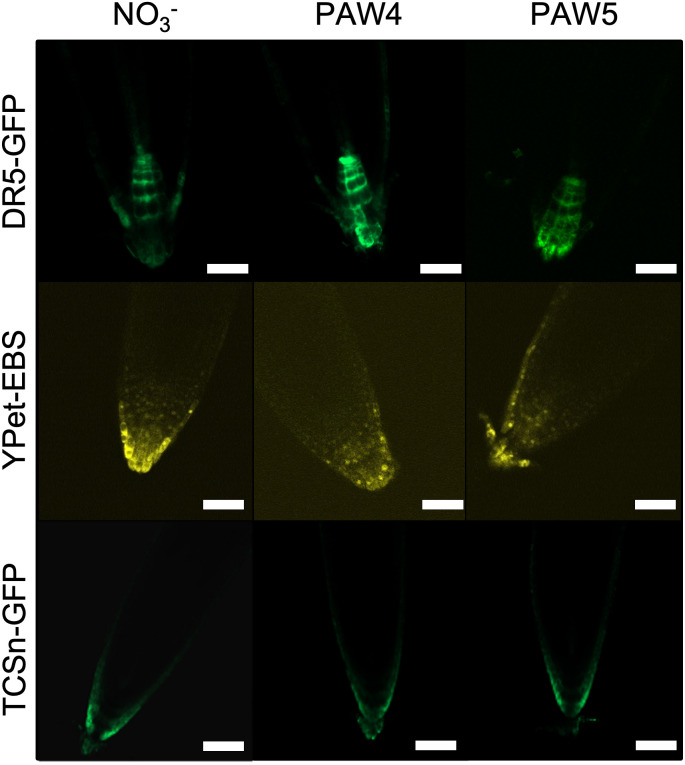
PAW-treatment does not alter the pattern of hormone response markers in roots. Three-day old Arabidopsis seedlings expressing fluorescent reporters for auxin (DR5::GFP), ethylene (EBS::Ypet) or cytokinin (TCSn::GFP) response pathways were transferred to N-free media supplemented with 2.4mM NO_3_^-^ (NO_3_^-^, control), PAW4 or PAW5. Root tips were imaged by confocal microscopy after 2 days of treatment. Images are representative of 5 replicate roots with similar patterns of fluorescence. Scale = 100 µm.

While seedlings treated with PAW4 exhibit increased root elongation, the underlying cause of this elongation remains unknown. Fluorescent markers were used to determine whether PAW treatment resulted in changes in plant hormone signaling in root tissue. Markers for auxin, ethylene, and cytokinin response were selected due to the role of these hormones in controlling root morphology under varying NO_3_^-^ levels [12,13,15]. The DR5::GFP marker is a transcriptional fusion of the synthetic DR5 promoter with GFP such that GFP accumulates in cells where auxin signaling is active [55]. A EBS::YPet marker was used to visualize ethylene response as the EBS promoter functions downstream of ethylene perception and signaling [57]. The TCSn::GFP marker was used to compare the response to cytokinin, as the TCSn promoter is responsive to that hormone [56,79]. Arabidopsis seedlings expressing each marker were treated with PAW4, PAW5, or the NO_3_^-^ control for two days and then imaged by confocal microscopy (Fig 4). All DR5::GFP treatments resulted in similar pattern of GFP fluorescence in the root meristematic region and in the innermost cell files of the root cap [55,80]. Similarly, all treated EBS::YPet roots showed similar patterns of YPet fluorescence concentrated at the root tip and the epidermis as previously reported [81,82]. Finally, no differences between treatments were detected with the TCSn::GFP lines, with fluorescence signal detected in the root epidermis approaching the apex of the root [79,83]. These results suggest that PAW4 and PAW5 treatments do not induce changes in auxin, ethylene, or cytokinin signaling pathways in the root compared to the NO_3_^-^ control.

### PAW treatment induced Nitrogen-responsive gene expression

Small increases in total root length were found for seedlings treated with PAW4 when compared to an equivalent NO_3_^-^ control. This led to the hypothesis that PAW treatment may alter gene expression. Wild-type three-day old seedlings were treated with PAW4 or an equivalent amount of NO_3_^-^ for 8 days, as described in previous experiments. RNA was extracted separately from shoots and roots from at least 20 seedlings per sample and sequenced using Next-Generation Sequencing. Sequence data was used to compare gene expression between PAW-treated tissues with the NO_3_^-^ controls. It should be noted that Principal Coordinate Analysis (PCA) of root samples indicated that one root sample each from PAW and control treatments did not cluster with the like samples (S3 Fig in S1 File). However, these samples were included due to the limited number of replicates.

**Fig 5:**
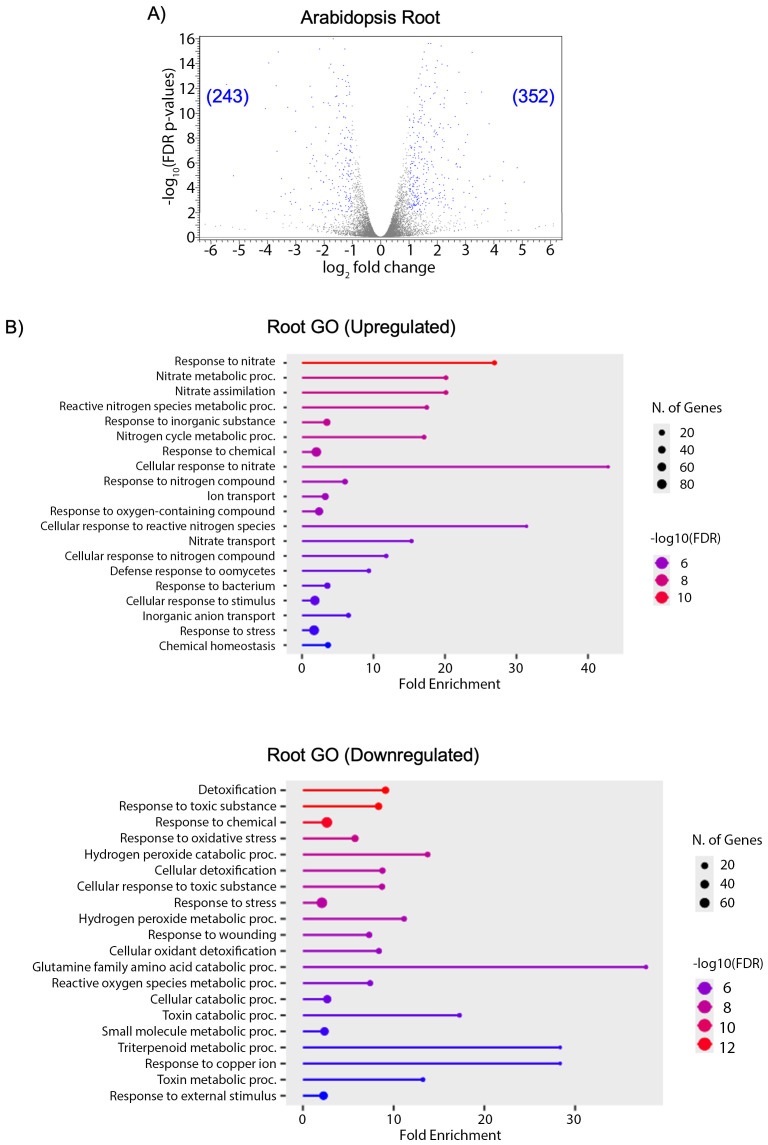
PAW treatment results in differential expression of genes associated with N and ROS responses in roots. Differential gene expression and functional enrichment analysis from RNA-seq data. Three-day old Arabidopsis seedlings were transferred to N-free media supplemented with PAW4 or 2.4mM NO_3_^-^ as a control. Roots were harvested one week later, and RNA was extracted and sequenced. A) Volcano plots displaying the distribution of differentially-expressed genes between PAW-treated and NO_3_^-^-treated controls in roots. Numbers represent the differentially expressed genes (blue dots) with p-values ≤ 0.01 and log2 fold change ≥ 1. B) Gene Ontology (GO) analysis of differentially expressed genes. Bar plots represent the top enriched GO terms in the biological processes category. Bar height indicates fold enrichment, the number of genes is indicated by size of the dots and color intensity reflects the statistical significance of enrichment (e.g., –log₁₀ *p*-value).

Differential expression analysis between PAW-treated tissues and NO_3_^-^ control samples included genes with FDR p-values ≤ 0.01 and at least a ≥ 2-fold change at a log_2_ transformation. A total of 352 genes were upregulated, while 243 downregulated in PAW-treated roots compared to the controls (Fig 5A). A gene ontology (GO) analysis was performed to identify biological pathways that may be altered by PAW exposure. Genes associated with nitrate assimilation and related metabolic pathways showed significant enrichment in PAW-treated roots when compared to the control (Fig 5B). Additionally, genes associated with responses to oxygen-containing compounds, such as ROS, showed approximately 50 genes with moderate fold enrichment (Fig 5B). Among the downregulated genes in roots, those associated with H_2_O_2_ metabolism and catabolism exhibited significantly lower fold enrichment and high -log_10_(FDR) values compared to the NO_3_^-^ control (Fig 5B). Notably, genes associated with glutamine catabolic processes, though a low proportion of the downregulated genes, still showed significance (Fig 5B). Glutamine is highly regulated in the plant and its control can also be varied by NH_4_^+^ availability and perception. Glutamine catabolism can result in glutamate, which can be recycled to form other N compounds [84].

**Fig 6:**
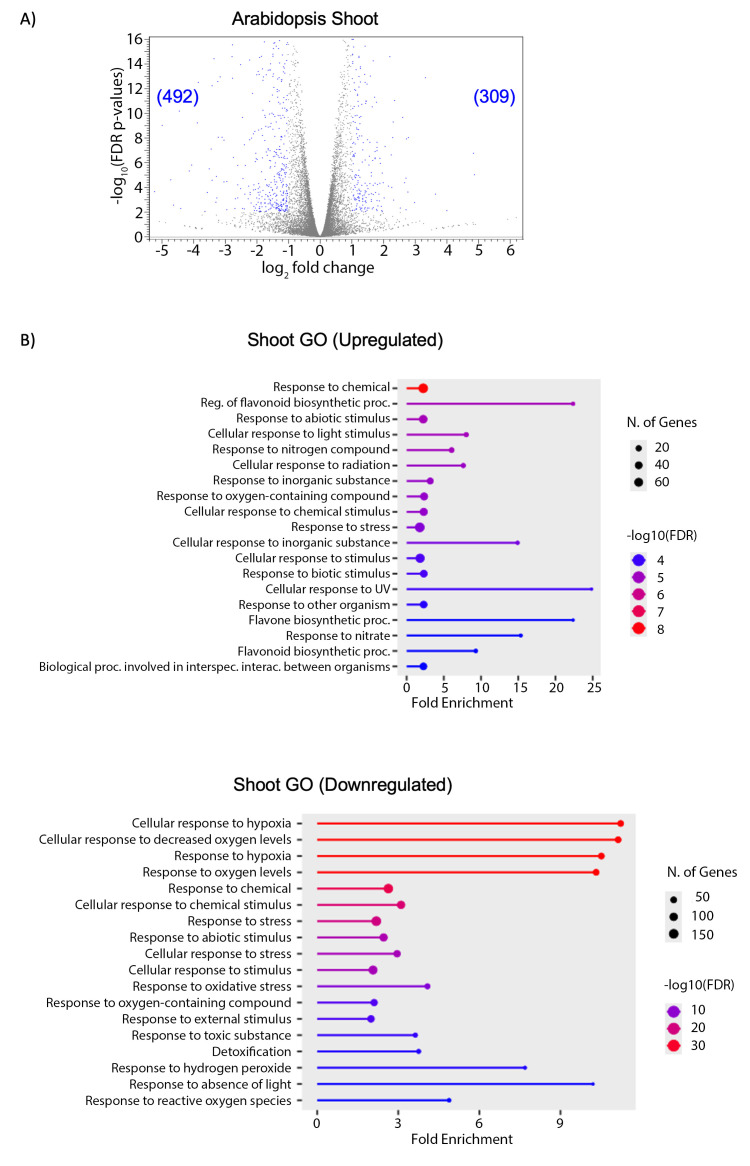
PAW treatment results in differential expression of genes associated with N and ROS responses in shoots. Differential gene expression and functional enrichment analysis from RNA-seq data. Three-day old Arabidopsis seedlings were transferred to N-free media supplemented with PAW4 or 2.4mM NO_3_^-^ as a control. Shoots were harvested one week later, and RNA was extracted and sequenced. A) Volcano plots displaying the distribution of differentially-expressed genes between PAW-treated and NO_3_^-^-treated controls in shoots. Numbers represent the number of differentially expressed genes (blue dots) with p-values ≤ 0.01 and log2 fold change ≥ 1. B) Gene Ontology (GO) analysis of differentially expressed genes in the shoot. Bar plots represent the top enriched GO terms in the biological processes category. Bar height indicates fold enrichment, the number of genes is indicated by size of the dots and color intensity reflects the statistical significance of enrichment (e.g., –log₁₀ *p*-value).

In the shoot samples, 309 genes were upregulated and 492 were downregulated in the PAW samples compared to the controls (Fig 6A). Upregulated genes were enriched in pathways associated with regulation of flavonoid biosynthesis and responses to nitrogen (Fig 6B). Flavonoids serve a variety of roles plants, including ROS scavenging [85]. This data suggests that seedlings treated with PAW exhibited an altered response to nitrate, potentially affecting uptake and assimilation rates. Furthermore, although PAW4 was intended to have limited ROS concentrations, the analysis suggests that PAW-treated plants may be responding to oxidative compounds. Among the downregulated genes, those associated with low O_2_ levels and stress responses were the most significantly impacted (Fig 6B).

### PAW4 is an effective fertilizer for soil-grown plants

While PAW treatment induces changes in root morphology in Arabidopsis 5-day old seedlings [48], little is known about the effects of prolonged treatment with PAW. The growth of Arabidopsis plants treated for up to 5 weeks with concentrated PAW4 (4.8 mM NO_3_^-^) was evaluated next because of its optimal performance compared to PAW5. Plants were regularly watered with diH_2_O and biweekly with a ¼ x Hoagland solution without N to provide all other essential nutrients. Plants were watered weekly with one of the treatment solutions, the NO_3_^-^ control or concentrated PAW4, as the only available source of N. After a 5-week growing period, plants were harvested to measure fresh and dry biomass of both shoot and root.

**Fig 7:**
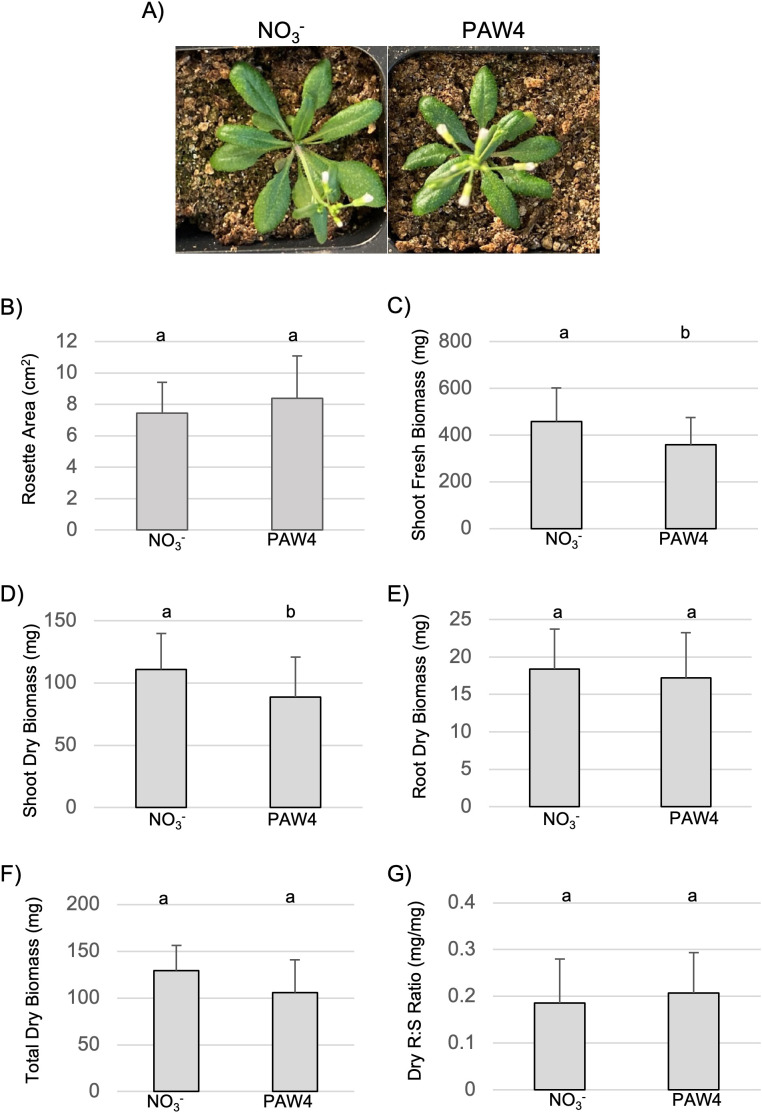
PAW is a good substitute for NO_3_^-^ fertilizer. Comparison between PAW and NO_3_^-^ treatment in soil-grown plants. A) Three-day old wild-type Arabidopsis seedlings were transplanted to soil without N and fertilized with either 4.8mM NO_3_^-^ or PAW4 (4.8mM NO_3_^-^) for 5 weeks. All other nutrients were provided. One representative image of plants after 5 weeks is shown. B) Rosette area of NO_3_^-^ and PAW-treated plants was measured from top images with ImageJ. C-G) Plants were harvested after 5 weeks of treatment and shoots and roots were used to measure shoot fresh biomass (C), shoot dry biomass (D), root dry biomass (E), Total dry biomass (F) and root:shoot ratio (G). N = 20 plants per treatment. Significance denotes p < 0.05 in Student’s T-test.

All plants developed healthy rosettes and inflorescences after 5 weeks (Fig 7A). No differences were detected in rosette area between PAW4-treated plants and those of the NO_3_^-^ control (Fig 7B). However, PAW4-treated plants showed a reduction in shoot fresh mass (Fig 7C) and shoot dry biomass (Fig 7D) when compared to plants treated with the NO_3_^-^ control. Interestingly, the PAW4-treated plants showed comparable dry root biomass to plants treated with the NO_3_^-^ control (Fig 7E). When combining both dry aboveground and root biomass, the total dry biomass was statistically similar between the two treatments (Fig 7F). Equivalent growth between the two treatments was also documented by their similar root:shoot ratio (Fig 7G). Despite reduced shoot dry biomass and similar root dry biomass of PAW4-treated plants, the root:shoot ratio suggests no difference of resource allocation between plants in either treatment. Together, these results suggest that PAW4 treatment and the NO_3_^-^ control yield comparable growth and overall dry biomass, supporting the use of PAW as a potential alternative to existing NO_3_^-^ fertilizers.

## Discussion

### PAW can be used as an alternative for N fertilizer

Nitrogen is a macronutrient for plants because it is a major component of nucleic acids and proteins. Plants primarily absorb N through their roots, sourcing the inorganic forms NO_3_^-^ and NH_4_^+^ from the soil environment [86]. Synthetic fertilizers containing NO_3_^-^ and NH_4_^+^ have become globally the most abundant type of N fertilizer over the last few decades [1]. Methods to produce these fertilizers are unsustainable, and they have an additional carbon footprint through transport and the supply chain [2]. There is an urgent need for the development of sustainable fertilizers that can meet growing agricultural demands, which PAW from non-thermal plasmas has the potential to meet [3]. However, diverse technologies for non-thermal plasma generation and water activation are still under development, and therefore, a unified standard in PAW production is not yet established [6,51].

PAW has been proposed as an effective source of N for plant growth [37,39,43,63,65,87]. Its most important contribution comes from its NO_3_^-^ content as substituting NO_3_^-^ solutions for PAW fully restores root growth (as compared to N-free water controls). PAW treatments with identical NO_3_^-^ content resulted in longer roots when compared to the NO_3_^-^ controls at the seedling stage indicating the presence of additional molecules with growth-promoting activity. These differences are certainly mild and unlikely due to H_2_O_2_ because this ROS was mostly inhibitory in controlled growth experiments in seedlings. We cannot rule out the possibility that the low levels of NH_4_^+^ detected in some PAWs may alter nitrogen signaling in ways that have not been accounted for. Our results are consistent with previous reports of enhanced germination and longer roots in PAW-treated Arabidopsis seedlings [48,62,64].

Transcriptome analysis showed that PAW treatment induced the expression of genes associated with N uptake and assimilation in roots. Specifically, genes from the NRT2 family such as NRT2.1, NRT2.6, and NRT2.4 were significantly upregulated. NRT2 family transporter proteins are characterized as high-affinity NO_3_^-^ transporters, often preferentially expressed in the root, and function in uptake from the soil [88–90]. NRT2.1 is a nitrate-inducible transporter that is highly expressed in the root when Arabidopsis is exposed to NO_3_^-^ [90], and post-translational control of NRT1.2 is critical for high affinity NO_3_- uptake [91]. The upregulation of these genes suggests that seedlings treated with PAW may be primed to more readily transport NO_3_^-^ from the soil environment. Furthermore, genes associated with NO_3_^-^ metabolism and assimilation were upregulated in these roots, which suggests increased perception and response to internal NO_3_^-^ levels in root cells [92,93]. Overall, the increased expression of these genes may contribute to the subtle root length increases noted at the seedling stage.

While this differential expression suggests PAW treatment improves NO_3_^-^ uptake and assimilation, these responses may only be short term. For instance, PAW4 treatment of mature plants in soil resulted in similar root growth as the NO_3_^-^ controls. This suggests that the enhanced root elongation effect of PAW detected in seedlings may be restricted to early developmental stages or to highly controlled environments such as the sterile Arabidopsis growth media. Long term (5-week) PAW4 treatment of plants in soil resulted in similar morphology and development compared to those watered with NO_3_^-^ control solution. Overall, there were no significant effects of PAW treatment in terms of total dry biomass and rosette area when compared to the NO_3_^-^ controls, indicating that plants respond to PAW in a similar manner as regular fertilizer in long-term experiments. Our results demonstrate that PAW is a sufficient fertilizer and a viable alternative to traditional NO_3_^-^ fertilizers for Arabidopsis seedlings and 5-week-old rosettes. Further studies focusing on the upregulation of genes associated with NO_3_^-^ uptake are needed to determine if treatment with trace ROS like those present in PAW4 may have benefits beyond nitrogen fertilization.

Many recent reports highlight dramatic differences in PAW-treated plants when compared to plants treated with tap water or deionized water alone. For example, certain PAW formulations increased barley fresh biomass up to 37% over a 4-week period (compared to a water control) [37], while other PAWs increased rice yield by 16.7% after a full growing season [65]. Maize showed a 13.1% dry biomass increase, though this was achieved through foliar sprays [43]. Pea plants also showed improved root and shoot growth, with increases up to 38% and 95% respectively, compared to a tap water control [39]. Most of these effects are likely attributed to the nitrogen content in PAW which is absent in the control treatment. In fact, these studies do not directly evaluate PAW’s potential as an alternative to traditional nitrogen fertilizers. The lack of an equivalent NO_3_^-^ control, coupled with the variability in chemical composition of PAWs from different plasma devices, make comparisons between different studies challenging. Moreover, very few studies specify the soil composition or tightly control for N content in soil. Most commercial soil or substrate formulations are likely amended with sufficient fertilizer for minimal plant growth, even if not specified in the label [94,95], which can seriously affect interpretation of PAW’s effects on plant growth. Our study used unamended commercial peatmoss, vermiculite, and perlite as raw materials to make soil substrates where NO_3_^-^ or PAW solutions were the only sources of plant-available N. Unlike previous PAW studies where plants in soil media were watered with PAW [36,39,63], plants did not survive beyond 3 weeks when watered with diH_2_O plus Hoagland minus N alone in this substrate. Future PAW studies should include NO_3_^-^ equivalent solutions to allow for comparisons between studies.

### ROS in PAW inhibits growth under ideal growth conditions

Previous reports have suggested that ROS in PAW contribute to growth enhancement beyond that of nitrate [39,65]. By generating PAW solutions that differed on the concentration of NO_3_^-^ and the presence of H_2_O_2_, this study evaluated the independent contributions of N and ROS. While high concentrations of exogenous ROS can be detrimental to plant health, low levels of ROS are thought to enhance plant growth [16,96]. These experiments showed that the primary root lengths of seedlings treated with both PAW types (1.75 and 2.4 mM NO_3_^-^) were shorter when H_2_O_2_ was present, indicating that even low concentrations of this ROS inhibited root growth. This was corroborated by controlled experiments using media containing as little as 0.05 mM of H_2_O_2_, which resulted in shorter roots in seedlings exposed to the same levels of N. These results suggest that PAW chemistries containing the lowest levels of H_2_O_2_ might be a better alternative for fertilizer substitution. Similar results previously reported that increased concentration of H_2_O_2_ in PAW result in oxidative stress, inhibition of seedling growth and shorter primary roots [46,48,64,97,98]. Arabidopsis specifically showed a 28% reduction in root elongation as concentrations of H_2_O_2_ increased after 7 days [48]. These results together with those reported in this study demonstrate that H_2_O_2_ does not provide any added benefit in PAW under optimal growing conditions.

A variety of ROS may accumulate within PAW [67,99], but are difficult to measure [38]. Theoretically, reactive nitrogen species (RNS) like peroxynitrite (ONOO^-^) or ROS like superoxide (O_2_^−^) may be present in the PAW [63,67,100], but at the current time the presence and concentrations of these species is unknown due to limitations in methods to measure these species cost-effectively. Fluorescent probes [70] were used as proxies to elucidate the effect of PAW4 and PAW5 on the plant root redox state. H_2_DCFDA responds generally to oxidation from endogenous ROS and works effectively in characterizing ROS response in Arabidopsis [69,70]. Both PAW4 and PAW5 treatments resulted in similar patterns of H_2_DCFDA staining when compared to their corresponding NO_3_^-^ or NO_3_^-^ plus H_2_O_2_ controls. We interpret this result as evidence that the main ROS in PAW is likely H_2_O_2_, and that no other ROS are present in PAW4 at concentrations that would elicit a plant response. This is further reinforced with PO1 staining, which specifically reports the accumulation of endogenous H_2_O_2_. From the 2-minute time point, both PAW 4 and 5 treatments and their corresponding controls containing NO_3_^-^ with or without H_2_O_2_ showed no difference in their response. The only response was observed in roots treated with a high (5 mM) concentration of H_2_O_2_. It is important to note that the NO_3_^-^ plus low (0.15 mM) concentration of H_2_O_2_ control showed no PO1 staining where the only difference between the two controls is the concentration of H_2_O_2_. This could be due to a difference in sensitivity between H_2_DCFDA and PO1, although other studies have not reported major sensitivity differences [101,102]. PO1 is a boronate-based probe, which means it can respond more specifically to H_2_O_2_ presence than H_2_DCFA. However, this type of probe can react slower than H_2_DCFDA [70,78]. If the peak of the intracellular H_2_O_2_ produced due to PAW treatment is transient, lasting only minutes, a slower probe like PO1 might report a weaker signal. It also suggests that the response to 0.15 mM H_2_O_2_ elicits a short-term response of endogenous ROS production where H_2_O_2_ is produced in low concentrations or is swiftly scavenged by antioxidants like catalase. This may explain why PAW treatment did not yield a strong response to ROS and PAW under these experimental conditions like the response found with H_2_DCFDA.

### PAW may confer stress resistance through priming with H_2_O_2_

PAW containing H_2_O_2_ may potentially provide stress protection to crop plants in the form of “priming” that lessens the impact of stressors [30–32]. Some research has shown that direct treatment of seeds with non-thermal plasma conferred benefits to seedlings undergoing osmotic and saline stress [62]. PAW treatment also enhanced cold-tolerance in tomato [87] and biotic stress resistance in grape vines [44]. It has been theorized that the RONS present in PAW are substantial enough to initiate the priming effect. This work focused on high temperature stress, which is becoming a more relevant stress for agriculture as global climate patterns continue changing at an accelerated rate [103]. A PAW5 pre-treatment seemed to protect Arabidopsis seedlings during a short-term high temperature stress, but PAW4 did not. This may be due to the presence of H_2_O_2_ in PAW5. It is important to note that a similar concentration of H_2_O_2_ was insufficient to provide the same benefit as PAW5, suggesting the presence of other molecular species in PAW5 that may enhance the priming effect. Overall, these results highlight the potential benefit of generating PAW with different levels of ROS to facilitate plant growth under stress and non-stress conditions.

Overall, this work demonstrated that PAW is an effective alternative for NO_3_^-^ fertilization in plants, but H_2_O_2_ concentrations must be evaluated carefully. Moreover, this work highlights the potential of H_2_O_2_-containing PAW solutions for providing protective effects in plants undergoing heat stress. Further studies with H_2_O_2_-containing PAW solutions in crop plants are needed to assess the potential of PAW as a priming treatment for stress resilience in field or greenhouse settings. One limitation of this work is that plants were tested only up to 4 weeks of Arabidopsis growth. Future research should address whether the nutrient-sufficient effect of PAW can be replicated later in development and under the energy requirements of plant reproduction. This work also highlights the importance of using equivalent NO_3_- controls to decouple the effects of NO_3_- and ROS in PAW experiments. Finally, continued development of PAW technologies to treat larger volumes of water are needed for large scale treatments of crop plants in soil and into mature developmental stages.

## Supporting information

S1 File S1 Fig. Quantification of RONS species in PAW. S2 Fig. PAW treatment resulted in variation in primary root length and lateral root density. S3 Fig. Principal Component Analysis of root tissue samples. (PDF)

## References

[1] Lassaletta L, Billen G, Grizzetti B, Anglade J, Garnier J. 50 year trends in nitrogen use efficiency of world cropping systems: the relationship between yield and nitrogen input to cropland. Environ Res Lett. 2014;9(10):105011. doi: 10.1088/1748-9326/9/10/105011

[2] Wang M, Khan MA, Mohsin I, Wicks J, Ip AH, Sumon KZ, et al. Can sustainable ammonia synthesis pathways compete with fossil-fuel based Haber–Bosch processes?. Energy Environ Sci. 2021;14(5):2535–48. doi: 10.1039/d0ee03808c

[3] Chen JG, Crooks RM, Seefeldt LC, Bren KL, Bullock RM, Darensbourg MY, et al. Beyond fossil fuel-driven nitrogen transformations. Science. 2018;360(6391):eaar6611. doi: 10.1126/science.aar6611 29798857 PMC6088796

[4] Bae JH, Lee H, Huh S-C, Park S. Nitric and nitrous acid formation in plasma-treated water: Decisive role of nitrogen oxides (NOx=1-3). Chemosphere. 2024;364:143105. doi: 10.1016/j.chemosphere.2024.143105 39153531

[5] Lietz AM, Kushner MJ. Air plasma treatment of liquid covered tissue: long timescale chemistry. J Phys D: Appl Phys. 2016;49(42):425204. doi: 10.1088/0022-3727/49/42/425204

[6] Robinson C, Stapelmann K. Plasma treating water for nitrate based nitrogen fertilizer - A review of recent device designs. Current Opinion in Green and Sustainable Chemistry. 2024;50:100978. doi: 10.1016/j.cogsc.2024.100978

[7] Zhang Y, Xia C, Zhang X, Sha Y, Feng G, Gao Q. Quantifying the relationships of soil properties and crop growth with yield in a NPK fertilizer application maize field. Computers and Electronics in Agriculture. 2022;198:107011. doi: 10.1016/j.compag.2022.107011

[8] Bloom AJ. The increasing importance of distinguishing among plant nitrogen sources. Curr Opin Plant Biol. 2015;25:10–6. doi: 10.1016/j.pbi.2015.03.002 25899331

[9] Krapp A. Plant nitrogen assimilation and its regulation: a complex puzzle with missing pieces. Curr Opin Plant Biol. 2015;25:115–22. doi: 10.1016/j.pbi.2015.05.010 26037390

[10] Yuan L, Loqué D, Kojima S, Rauch S, Ishiyama K, Inoue E, et al. The organization of high-affinity ammonium uptake in Arabidopsis roots depends on the spatial arrangement and biochemical properties of AMT1-type transporters. Plant Cell. 2007;19(8):2636–52. doi: 10.1105/tpc.107.052134 17693533 PMC2002620

[11] Ludewig U, Neuhäuser B, Dynowski M. Molecular mechanisms of ammonium transport and accumulation in plants. FEBS Lett. 2007;581(12):2301–8. doi: 10.1016/j.febslet.2007.03.034 17397837

[12] Bouguyon E, Gojon A, Nacry P. Nitrate sensing and signaling in plants. Semin Cell Dev Biol. 2012;23(6):648–54. doi: 10.1016/j.semcdb.2012.01.004 22273693

[13] Bouguyon E, Brun F, Meynard D, Kubeš M, Pervent M, Leran S, et al. Multiple mechanisms of nitrate sensing by Arabidopsis nitrate transceptor NRT1.1. Nat Plants. 2015;1:15015. doi: 10.1038/nplants.2015.15 27246882

[14] Maghiaoui A, Bouguyon E, Cuesta C, Perrine-Walker F, Alcon C, Krouk G, et al. The Arabidopsis NRT1.1 transceptor coordinately controls auxin biosynthesis and transport to regulate root branching in response to nitrate. J Exp Bot. 2020;71(15):4480–94. doi: 10.1093/jxb/eraa242 32428238

[15] Krouk G, Lacombe B, Bielach A, Perrine-Walker F, Malinska K, Mounier E, et al. Nitrate-regulated auxin transport by NRT1.1 defines a mechanism for nutrient sensing in plants. Dev Cell. 2010;18(6):927–37. doi: 10.1016/j.devcel.2010.05.008 20627075

[16] Singh R, Singh S, Parihar P, Mishra RK, Tripathi DK, Singh VP, et al. Reactive Oxygen Species (ROS): Beneficial Companions of Plants’ Developmental Processes. Front Plant Sci. 2016;7:1299. doi: 10.3389/fpls.2016.01299 27729914 PMC5037240

[17] Kliebenstein DJ, Monde RA, Last RL. Superoxide dismutase in Arabidopsis: an eclectic enzyme family with disparate regulation and protein localization. Plant Physiol. 1998;118(2):637–50. doi: 10.1104/pp.118.2.637 9765550 PMC34840

[18] Pilon M, Ravet K, Tapken W. The biogenesis and physiological function of chloroplast superoxide dismutases. Biochim Biophys Acta. 2011;1807(8):989–98. doi: 10.1016/j.bbabio.2010.11.002 21078292

[19] Tuzet A, Rahantaniaina M-S, Noctor G. Analyzing the Function of Catalase and the Ascorbate-Glutathione Pathway in H2O2 Processing: Insights from an Experimentally Constrained Kinetic Model. Antioxid Redox Signal. 2019;30(9):1238–68. doi: 10.1089/ars.2018.7601 30044135

[20] Bueso E, Alejandro S, Carbonell P, Perez-Amador MA, Fayos J, Bellés JM, et al. The lithium tolerance of the Arabidopsis cat2 mutant reveals a cross-talk between oxidative stress and ethylene. Plant J. 2007;52(6):1052–65. doi: 10.1111/j.1365-313X.2007.03305.x 17931347

[21] Dvořák P, Krasylenko Y, Ovečka M, Basheer J, Zapletalová V, Šamaj J, et al. In vivo light-sheet microscopy resolves localisation patterns of FSD1, a superoxide dismutase with function in root development and osmoprotection. Plant Cell Environ. 2021;44(1):68–87. doi: 10.1111/pce.13894 32974958

[22] Tsukagoshi H. Control of root growth and development by reactive oxygen species. Curr Opin Plant Biol. 2016;29:57–63. doi: 10.1016/j.pbi.2015.10.012 26724502

[23] Chapman JM, Muhlemann JK, Gayomba SR, Muday GK. RBOH-Dependent ROS Synthesis and ROS Scavenging by Plant Specialized Metabolites To Modulate Plant Development and Stress Responses. Chem Res Toxicol. 2019;32(3):370–96. doi: 10.1021/acs.chemrestox.9b00028 30781949 PMC6857786

[24] Voothuluru P, Mäkelä P, Zhu J, Yamaguchi M, Cho I-J, Oliver MJ, et al. Apoplastic Hydrogen Peroxide in the Growth Zone of the Maize Primary Root. Increased Levels Differentially Modulate Root Elongation Under Well-Watered and Water-Stressed Conditions. Front Plant Sci. 2020;11:392. doi: 10.3389/fpls.2020.00392 32373139 PMC7186474

[25] Karpinska B, Foyer CH. Superoxide signalling and antioxidant processing in the plant nucleus. J Exp Bot. 2024;75(15):4599–610. doi: 10.1093/jxb/erae090 38460122 PMC11317529

[26] Tsukagoshi H, Busch W, Benfey PN. Transcriptional regulation of ROS controls transition from proliferation to differentiation in the root. Cell. 2010;143(4):606–16. doi: 10.1016/j.cell.2010.10.020 21074051

[27] Yao Y, He RJ, Xie QL, Zhao XH, Deng XM, He JB, et al. Ethylene response factor 74 (ERF74) plays an essential role in controlling a respiratory burst oxidase homolog D (RbohD)‐dependent mechanism in response to different stresses in Arabidopsis. New Phytologist. 2017;213(4):1667–81. 28164334 10.1111/nph.14278

[28] Guo F-Q, Okamoto M, Crawford NM. Identification of a plant nitric oxide synthase gene involved in hormonal signaling. Science. 2003;302(5642):100–3. doi: 10.1126/science.1086770 14526079

[29] Gilroy S, Białasek M, Suzuki N, Górecka M, Devireddy AR, Karpiński S, et al. ROS, Calcium, and Electric Signals: Key Mediators of Rapid Systemic Signaling in Plants. Plant Physiol. 2016;171(3):1606–15. doi: 10.1104/pp.16.00434 27208294 PMC4936577

[30] Mittler R, Blumwald E. The roles of ROS and ABA in systemic acquired acclimation. Plant Cell. 2015;27(1):64–70. doi: 10.1105/tpc.114.133090 25604442 PMC4330577

[31] Rodríguez-Ruiz M, Zuccarelli R, Palma JM, Corpas FJ, Freschi L. Biotechnological application of nitric oxide and hydrogen peroxide in plants. In: Gupta DK, Palma JM, Corpas FJ. Nitric oxide and hydrogen peroxide signaling in higher plants. Cham: Springer International Publishing. 2019. 245–70.

[32] Savvides A, Ali S, Tester M, Fotopoulos V. Chemical Priming of Plants Against Multiple Abiotic Stresses: Mission Possible?. Trends Plant Sci. 2016;21(4):329–40. doi: 10.1016/j.tplants.2015.11.003 26704665

[33] Zhu CQ, Hu WJ, Cao XC, Zhu LF, Bai ZG, Liang QD, et al. Hydrogen peroxide alleviates P starvation in rice by facilitating P remobilization from the root cell wall. J Plant Physiol. 2019;240:153003. doi: 10.1016/j.jplph.2019.153003 31279219

[34] Chen S, Liu H, Yangzong Z, Gardea-Torresdey JL, White JC, Zhao L. Seed Priming with Reactive Oxygen Species-Generating Nanoparticles Enhanced Maize Tolerance to Multiple Abiotic Stresses. Environ Sci Technol. 2023;57(48):19932–41. doi: 10.1021/acs.est.3c07339 37975618

[35] Wang X, Ge J, He M, Li Q, Cai J, Zhou Q, et al. Enhancing crop resilience: Understanding the role of drought priming in wheat stress response. Field Crops Research. 2023;302:109083.

[36] Lamichhane P, Veerana M, Lim JS, Mumtaz S, Shrestha B, Kaushik NK, et al. Low-Temperature Plasma-Assisted Nitrogen Fixation for Corn Plant Growth and Development. Int J Mol Sci. 2021;22(10):5360. doi: 10.3390/ijms22105360 34069725 PMC8161386

[37] Ndiffo Yemeli GB, Švubová R, Kostolani D, Kyzek S, Machala Z. The effect of water activated by nonthermal air plasma on the growth of farm plants: Case of maize and barley. Plasma Processes & Polymers. 2021;18(1). doi: 10.1002/ppap.202000205

[38] Ran C, Zhou X, Wang Z, Liu K, Ostrikov K (Ken). Ultralong-lasting plasma-activated water: production and control mechanisms. Plasma Sources Sci Technol. 2024;33(1):015009. doi: 10.1088/1361-6595/ad1b6c

[39] Rathore V, Tiwari BS, Nema SK. Treatment of Pea Seeds with Plasma Activated Water to Enhance Germination, Plant Growth, and Plant Composition. Plasma Chem Plasma Process. 2022;42(1):109–29. doi: 10.1007/s11090-021-10211-5

[40] Wang Y, Wang S, Sun J, Dai H, Zhang B, Xiang W, et al. Nanobubbles promote nutrient utilization and plant growth in rice by upregulating nutrient uptake genes and stimulating growth hormone production. Sci Total Environ. 2021;800:149627. doi: 10.1016/j.scitotenv.2021.149627 34426308

[41] Adhikari B, Adhikari M, Ghimire B, Park G, Choi EH. Cold Atmospheric Plasma-Activated Water Irrigation Induces Defense Hormone and Gene expression in Tomato seedlings. Sci Rep. 2019;9(1):16080. doi: 10.1038/s41598-019-52646-z 31695109 PMC6834632

[42] Kučerová K, Henselová M, Slováková Ľ, Bačovčinová M, Hensel K. Effect of Plasma Activated Water, Hydrogen Peroxide, and Nitrates on Lettuce Growth and Its Physiological Parameters. Applied Sciences. 2021;11(5).

[43] Škarpa P, Klofáč D, Krčma F, Šimečková J, Kozáková Z. Effect of Plasma Activated Water Foliar Application on Selected Growth Parameters of Maize (Zea mays L.). Water. 2020;12(12).

[44] Zambon Y, Contaldo N, Laurita R, Várallyay E, Canel A, Gherardi M, et al. Plasma activated water triggers plant defence responses. Sci Rep. 2020;10(1):19211. doi: 10.1038/s41598-020-76247-3 33154510 PMC7644721

[45] Cui D, Yin Y, Li H, Hu X, Zhuang J, Ma R, et al. Comparative transcriptome analysis of atmospheric pressure cold plasma enhanced early seedling growth in Arabidopsis thaliana. Plasma Sci Technol. 2021;23(8):085502. doi: 10.1088/2058-6272/ac0686

[46] Cui D, Yin Y, Sun H, Wang X, Zhuang J, Wang L, et al. Regulation of cellular redox homeostasis in Arabidopsis thaliana seedling by atmospheric pressure cold plasma-generated reactive oxygen/nitrogen species. Ecotoxicol Environ Saf. 2022;240:113703. doi: 10.1016/j.ecoenv.2022.113703 35659700

[47] Cortese E, Settimi AG, Pettenuzzo S, Cappellin L, Galenda A, Famengo A, et al. Plasma-activated water triggers rapid and sustained cytosolic Ca2+ elevations in Arabidopsis thaliana. Plants. 2021;10(11). 10.3390/plants10112516 PMC8622995 34834879

[48] Ka DH, Priatama RA, Park JY, Park SJ, Kim SB, Lee IA, et al. Plasma-Activated Water Modulates Root Hair Cell Density via Root Developmental Genes in Arabidopsis thaliana L. Applied Sciences. 2021;11(5):2240. doi: 10.3390/app11052240

[49] Byrns B, Wooten D, Lindsay A, Shannon S. A VHF driven coaxial atmospheric air plasma: electrical and optical characterization. J Phys D: Appl Phys. 2012;45(19):195204. doi: 10.1088/0022-3727/45/19/195204

[50] Lindsay A, Byrns B, King W, Andhvarapou A, Fields J, Knappe D, et al. Fertilization of radishes, tomatoes, and marigolds using a large-volume atmospheric glow discharge. Plasma Chemistry and Plasma Processing. 2014;34(6):1271–90.

[51] Ranieri P, Sponsel N, Kizer J, Rojas‐Pierce M, Hernández R, Gatiboni L, et al. Plasma agriculture: Review from the perspective of the plant and its ecosystem. Plasma Processes & Polymers. 2020;18(1). doi: 10.1002/ppap.202000162

[52] Rathore V, Nema SK. Optimization of process parameters to generate plasma activated water and study of physicochemical properties of plasma activated solutions at optimum condition. Journal of Applied Physics. 2021;129(8).

[53] Zhang H, Rubab M, Chen M, Gao J, Sun Q, Xia Q, et al. Study on the detection of active components in plasma-activated water and its storage stability. CyTA-Journal of Food. 2024;22(1):2386417.

[54] Julák J, Hujacová A, Scholtz V, Khun J, Holada K. Contribution to the chemistry of plasma-activated water. Plasma Physics Reports. 2018;44(1):125–36.

[55] Ulmasov T, Murfett J, Hagen G, Guilfoyle TJ. Aux/IAA proteins repress expression of reporter genes containing natural and highly active synthetic auxin response elements. Plant Cell. 1997;9(11):1963–71. doi: 10.1105/tpc.9.11.1963 9401121 PMC157050

[56] Zürcher E, Tavor-Deslex D, Lituiev D, Enkerli K, Tarr PT, Müller B. A robust and sensitive synthetic sensor to monitor the transcriptional output of the cytokinin signaling network in planta. Plant Physiol. 2013;161(3):1066–75. doi: 10.1104/pp.112.211763 23355633 PMC3585579

[57] Fernandez‐Moreno J, Stepanova AN. Monitoring Ethylene in Plants: Genetically Encoded Reporters and Biosensors. Small Methods. 2019;4(8). doi: 10.1002/smtd.201900260

[58] Schindelin J, Arganda-Carreras I, Frise E, Kaynig V, Longair M, Pietzsch T, et al. Fiji: an open-source platform for biological-image analysis. Nat Methods. 2012;9(7):676–82. doi: 10.1038/nmeth.2019 22743772 PMC3855844

[59] Ge SX, Jung D, Yao R. ShinyGO: a graphical gene-set enrichment tool for animals and plants. Bioinformatics. 2019;36(8):2628–9. doi: 10.1093/bioinformatics/btz931 31882993 PMC7178415

[60] Agbangba CE, Sacla Aide E, Honfo H, Glèlè Kakai R. On the use of post-hoc tests in environmental and biological sciences: A critical review. Heliyon. 2024;10(3):e25131. doi: 10.1016/j.heliyon.2024.e25131 39668858 PMC11637079

[61] Boer MD, Santos Teixeira J, Ten Tusscher KH. Modeling of Root Nitrate Responses Suggests Preferential Foraging Arises From the Integration of Demand, Supply and Local Presence Signals. Front Plant Sci. 2020;11:708. doi: 10.3389/fpls.2020.00708 32536935 PMC7268170

[62] Bafoil M, Le Ru A, Merbahi N, Eichwald O, Dunand C, Yousfi M. New insights of low-temperature plasma effects on germination of three genotypes of Arabidopsis thaliana seeds under osmotic and saline stresses. Sci Rep. 2019;9(1):8649. doi: 10.1038/s41598-019-44927-4 31209339 PMC6572809

[63] Kučerová K, Henselová M, Slováková Ľ, Hensel K. Effects of plasma activated water on wheat: Germination, growth parameters, photosynthetic pigments, soluble protein content, and antioxidant enzymes activity. Plasma Processes & Polymers. 2019;16(3). doi: 10.1002/ppap.201800131

[64] Cui D, Yin Y, Wang J, Wang Z, Ding H, Ma R, et al. Research on the Physio-Biochemical Mechanism of Non-Thermal Plasma-Regulated Seed Germination and Early Seedling Development in Arabidopsis. Front Plant Sci. 2019;10:1322. doi: 10.3389/fpls.2019.01322 31781132 PMC6857620

[65] Rashid M, Rashid MM, Reza MA, Talukder MR. Combined Effects of Air Plasma Seed Treatment and Foliar Application of Plasma Activated Water on Enhanced Paddy Plant Growth and Yield. Plasma Chem Plasma Process. 2021;41(4):1081–99. doi: 10.1007/s11090-021-10179-2

[66] Gierczik K, Vukušić T, Kovács L, Székely A, Szalai G, Milošević S, et al. Plasma‐activated water to improve the stress tolerance of barley. Plasma Processes & Polymers. 2020;17(3). doi: 10.1002/ppap.201900123

[67] Iseni S, Bruggeman PJ, Weltmann K-D, Reuter S. Nitrogen metastable (N2(A3Σu+)) in a cold argon atmospheric pressure plasma jet: Shielding and gas composition. Applied Physics Letters. 2016;108(18). doi: 10.1063/1.4948535

[68] Traylor MJ, Pavlovich MJ, Karim S, Hait P, Sakiyama Y, Clark DS, et al. Long-term antibacterial efficacy of air plasma-activated water. J Phys D: Appl Phys. 2011;44(47):472001. doi: 10.1088/0022-3727/44/47/472001

[69] Kuběnová LA, Haberland JA, Dvořák PA, Šamaj JA, Ovečka MA. Spatiotemporal distribution of reactive oxygen species production, delivery, and use in Arabidopsis root hairs. 2023. 10.1093/plphys/kiad484 PMC10663114 37666000

[70] Martin RE, Postiglione AE, Muday GK. Reactive oxygen species function as signaling molecules in controlling plant development and hormonal responses. Curr Opin Plant Biol. 2022;69:102293. doi: 10.1016/j.pbi.2022.102293 36099672 PMC10475289

[71] Dickinson BC, Huynh C, Chang CJ. A palette of fluorescent probes with varying emission colors for imaging hydrogen peroxide signaling in living cells. J Am Chem Soc. 2010;132(16):5906–15. doi: 10.1021/ja1014103 20361787 PMC2862989

[72] Shin R, Berg RH, Schachtman DP. Reactive oxygen species and root hairs in Arabidopsis root response to nitrogen, phosphorus and potassium deficiency. Plant Cell Physiol. 2005;46(8):1350–7. doi: 10.1093/pcp/pci145 15946982

[73] Zhu C, Yang N, Guo Z, Qian M, Gan L. An ethylene and ROS-dependent pathway is involved in low ammonium-induced root hair elongation in Arabidopsis seedlings. Plant Physiol Biochem. 2016;105:37–44. doi: 10.1016/j.plaphy.2016.04.002 27074220

[74] Bai J, Rodriguez AM, Melendez JA, Cederbaum AI. Overexpression of catalase in cytosolic or mitochondrial compartment protects HepG2 cells against oxidative injury. J Biol Chem. 1999;274(37):26217–24. doi: 10.1074/jbc.274.37.26217 10473575

[75] Bi Y, Chen W, Zhang W, Zhou Q, Yun L, Xing D. Production of reactive oxygen species, impairment of photosynthetic function and dynamic changes in mitochondria are early events in cadmium-induced cell death in Arabidopsis thaliana. Biol Cell. 2009;101(11):629–43. doi: 10.1042/BC20090015 19453296

[76] Guler NS, Pehlivan N. Exogenous low-dose hydrogen peroxide enhances drought tolerance of soybean (Glycine max L.) through inducing antioxidant system. Acta Biol Hung. 2016;67(2):169–83. doi: 10.1556/018.67.2016.2.5 27165528

[77] Tognetti VB, Bielach A, Hrtyan M. Redox regulation at the site of primary growth: auxin, cytokinin and ROS crosstalk. Plant Cell Environ. 2017;40(11):2586–605. doi: 10.1111/pce.13021 28708264

[78] Winterbourn CC. The challenges of using fluorescent probes to detect and quantify specific reactive oxygen species in living cells. Biochim Biophys Acta. 2014;1840(2):730–8. doi: 10.1016/j.bbagen.2013.05.004 23665586

[79] Liu J, Müller B. Imaging TCSn::GFP, a synthetic cytokinin reporter, in Arabidopsis thaliana. In: Kleine-Vehn J, Sauer M. Plant Hormones: Methods and Protocols. New York, NY: Springer New York. 2017. 81–90. 10.1007/978-1-4939-6469-7_9 27864760

[80] Vicente-Agullo F, Rigas S, Desbrosses G, Dolan L, Hatzopoulos P, Grabov A. Potassium carrier TRH1 is required for auxin transport in Arabidopsis roots. Plant J. 2004;40(4):523–35. doi: 10.1111/j.1365-313X.2004.02230.x 15500468

[81] Herrera-Rodríguez MB, Camacho-Cristóbal JJ, Barrero-Rodríguez R, Rexach J, Navarro-Gochicoa MT, González-Fontes A. Crosstalk of Cytokinin with Ethylene and Auxin for Cell Elongation Inhibition and Boron Transport in Arabidopsis Primary Root under Boron Deficiency. Plants (Basel). 2022;11(18):2344. doi: 10.3390/plants11182344 36145745 PMC9504276

[82] Stepanova AN, Yun J, Likhacheva AV, Alonso JM. Multilevel interactions between ethylene and auxin in Arabidopsis roots. Plant Cell. 2007;19(7):2169–85. doi: 10.1105/tpc.107.052068 17630276 PMC1955696

[83] Steiner E, Israeli A, Gupta R, Shwartz I, Nir I, Leibman-Markus M, et al. Characterization of the cytokinin sensor TCSv2 in arabidopsis and tomato. Plant Methods. 2020;16(1):152. doi: 10.1186/s13007-020-00694-2 33292327 PMC7670716

[84] Miflin BJ, Habash DZ. The role of glutamine synthetase and glutamate dehydrogenase in nitrogen assimilation and possibilities for improvement in the nitrogen utilization of crops. J Exp Bot. 2002;53(370):979–87. doi: 10.1093/jexbot/53.370.979 11912240

[85] Di Ferdinando M, Brunetti C, Fini A, Tattini M. Abiotic stress responses in plants. Springer New York, NY, USA. 2012.

[86] Hachiya T, Sakakibara H. Interactions between nitrate and ammonium in their uptake, allocation, assimilation, and signaling in plants. J Exp Bot. 2017;68(10):2501–12. doi: 10.1093/jxb/erw449 28007951

[87] Li K, Zhong C, Shi Q, Bi H, Gong B. Cold plasma seed treatment improves chilling resistance of tomato plants through hydrogen peroxide and abscisic acid signaling pathway. Free Radic Biol Med. 2021;172:286–97. doi: 10.1016/j.freeradbiomed.2021.06.011 34139310

[88] Zhuo M, Sakuraba Y, Yanagisawa S. Dof1.7 and NIGT1 transcription factors mediate multilayered transcriptional regulation for different expression patterns of NITRATE TRANSPORTER2 genes under nitrogen deficiency stress. New Phytol. 2024;242(5):2132–47. doi: 10.1111/nph.19695 38523242

[89] Orsel M, Krapp A, Daniel-Vedele F. Analysis of the NRT2 nitrate transporter family in Arabidopsis. Structure and gene expression. Plant Physiol. 2002;129(2):886–96. doi: 10.1104/pp.005280 12068127 PMC161709

[90] Okamoto M, Vidmar JJ, Glass ADM. Regulation of NRT1 and NRT2 gene families of Arabidopsis thaliana: responses to nitrate provision. Plant Cell Physiol. 2003;44(3):304–17. doi: 10.1093/pcp/pcg036 12668777

[91] Laugier E, Bouguyon E, Mauriès A, Tillard P, Gojon A, Lejay L. Regulation of high-affinity nitrate uptake in roots of Arabidopsis depends predominantly on posttranscriptional control of the NRT2.1/NAR2.1 transport system. Plant Physiol. 2012;158(2):1067–78. doi: 10.1104/pp.111.188532 22158677 PMC3271743

[92] Wang R, Guegler K, LaBrie ST, Crawford NM. Genomic analysis of a nutrient response in Arabidopsis reveals diverse expression patterns and novel metabolic and potential regulatory genes induced by nitrate. Plant Cell. 2000;12(8):1491–509. doi: 10.1105/tpc.12.8.1491 10948265 PMC149118

[93] Zayed O, Hewedy OA, Abdelmoteleb A, Ali M, Youssef MS, Roumia AF, et al. Nitrogen journey in plants: from uptake to metabolism, stress response, and microbe interaction. Biomolecules. 2023;13(10). 10.3390/biom13101443 PMC10605003 37892125

[94] Williams KA. Greenhouse fertilization of the future: imagine the possibilities. NC Flower Growers’ Bulletin. 1995;40(2):9–13.

[95] Dalling JW, Winter K, Andersen KM, Turner BL. Artefacts of the pot environment on soil nutrient availability: implications for the interpretation of ecological studies. Plant Ecol. 2013;214(2):329–38. doi: 10.1007/s11258-013-0172-3

[96] Berrios L, Rentsch JD. Linking Reactive Oxygen Species (ROS) to Abiotic and Biotic Feedbacks in Plant Microbiomes: The Dose Makes the Poison. Int J Mol Sci. 2022;23(8):4402. doi: 10.3390/ijms23084402 35457220 PMC9030523

[97] Zhou L, Hou H, Yang T, Lian Y, Sun Y, Bian Z, et al. Exogenous hydrogen peroxide inhibits primary root gravitropism by regulating auxin distribution during Arabidopsis seed germination. Plant Physiol Biochem. 2018;128:126–33. doi: 10.1016/j.plaphy.2018.05.014 29775864

[98] Panngom K, Chuesaard T, Tamchan N, Jiwchan T, Srikongsritong K, Park G. Comparative assessment for the effects of reactive species on seed germination, growth and metabolisms of vegetables. Scientia Horticulturae. 2018;227:85–91. doi: 10.1016/j.scienta.2017.09.026

[99] Gorbanev Y, O’Connell D, Chechik V. Non-Thermal Plasma in Contact with Water: The Origin of Species. Chemistry. 2016;22(10):3496–505. doi: 10.1002/chem.201503771 26833560 PMC4797710

[100] Tachibana K, Nakamura T. Comparative study of discharge schemes for production rates and ratios of reactive oxygen and nitrogen species in plasma activated water. J Phys D: Appl Phys. 2019;52(38):385202. doi: 10.1088/1361-6463/ab2529

[101] Gayomba SR, Muday GK. Flavonols regulate root hair development by modulating accumulation of reactive oxygen species in the root epidermis. Development. 2020;147(8):dev185819. doi: 10.1242/dev.185819 32179566

[102] Watkins JM, Chapman JM, Muday GK. Abscisic Acid-Induced Reactive Oxygen Species Are Modulated by Flavonols to Control Stomata Aperture. Plant Physiol. 2017;175(4):1807–25. doi: 10.1104/pp.17.01010 29051198 PMC5717730

[103] Ainsworth EA, Ort DR. How do we improve crop production in a warming world?. Plant Physiol. 2010;154(2):526–30. doi: 10.1104/pp.110.161349 20921178 PMC2949002

