# Supplementary material for: Non-thermal plasma activated water is an effective nitrogen fertilizer alternative for Arabidopsis thaliana
Source: PLoS One. 2025 Sep 8;20(9):e0327091. doi: 10.1371/journal.pone.0327091 (PMC12416708; doi:10.1371/journal.pone.0327091)
Supplement: S1 File — (PDF) [file pone.0327091.s001.pdf]

Non-Thermal Plasma Activated Water is an Effective Nitrogen Fertilizer Alternative for *Arabidopsis thaliana*

SUPPORTING INFORMATION

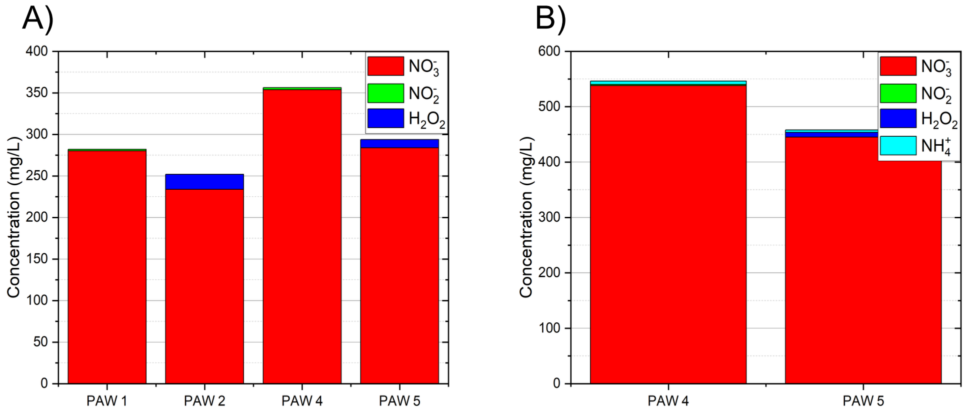

S1 Figure. Quantification of RONS species in PAW.

A) Representative concentrations of reactive nitrogen oxygen species (RONS) measured in the plasma activated water (PAW1-4) directly after treatment, prior to buffering and dilution. All PAWs were produced using the atmospheric pressure radio frequency plasma device as described in the methods. The concentrations were measured with Supelco test kits via optical absorbance spectroscopy. Measured absorbance values were compared to those obtained from stock solution based standard curves to obtain values in mg/L. B) Representative concentrations of reactive nitrogen oxygen species (RONS) for PAW4 and PAW5 including ammonium. Measurements were taken directly after treatment, prior to buffering and dilution. Ammonium measurements were carried out similarly to the other colorimetrics, as previously mentioned.

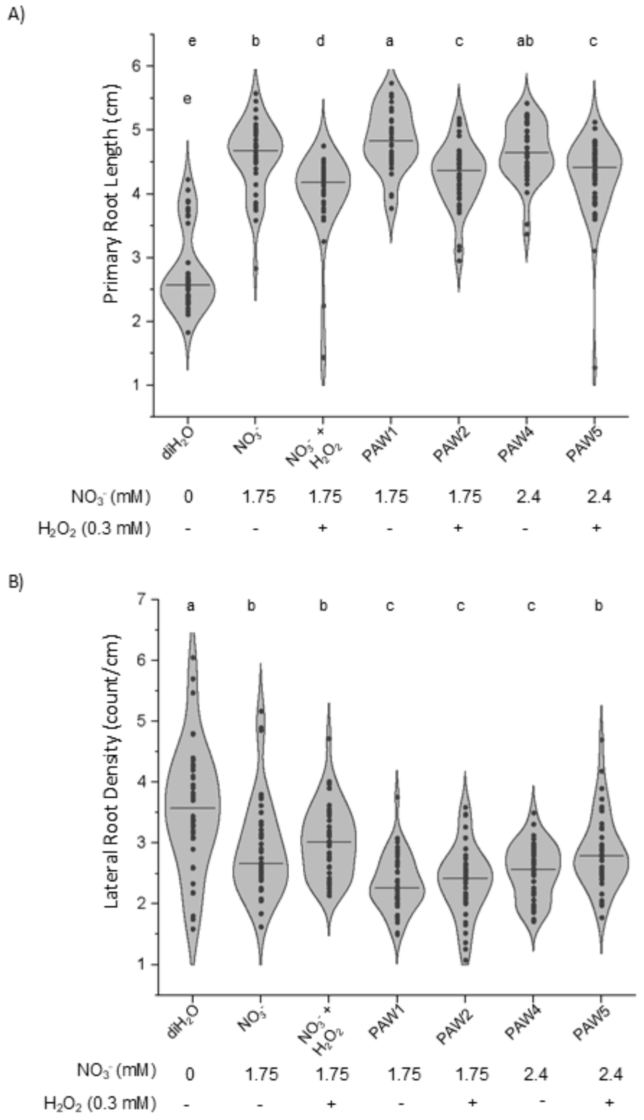

S2 Figure. PAW treatment resulted in variation in primary root length and lateral root density

Three-day old Arabidopsis seedlings were transferred to N-free media supplemented with diH_2O (control), 1.75 mM or 2.4 mM NO_3^-, with or without H_2O_2 for 5 days. Primary root length (A) and lateral root density (B) were quantified in Fiji. A one-way ANOVA with Tukey's multiple comparison test was performed. N= 40 seedlings.

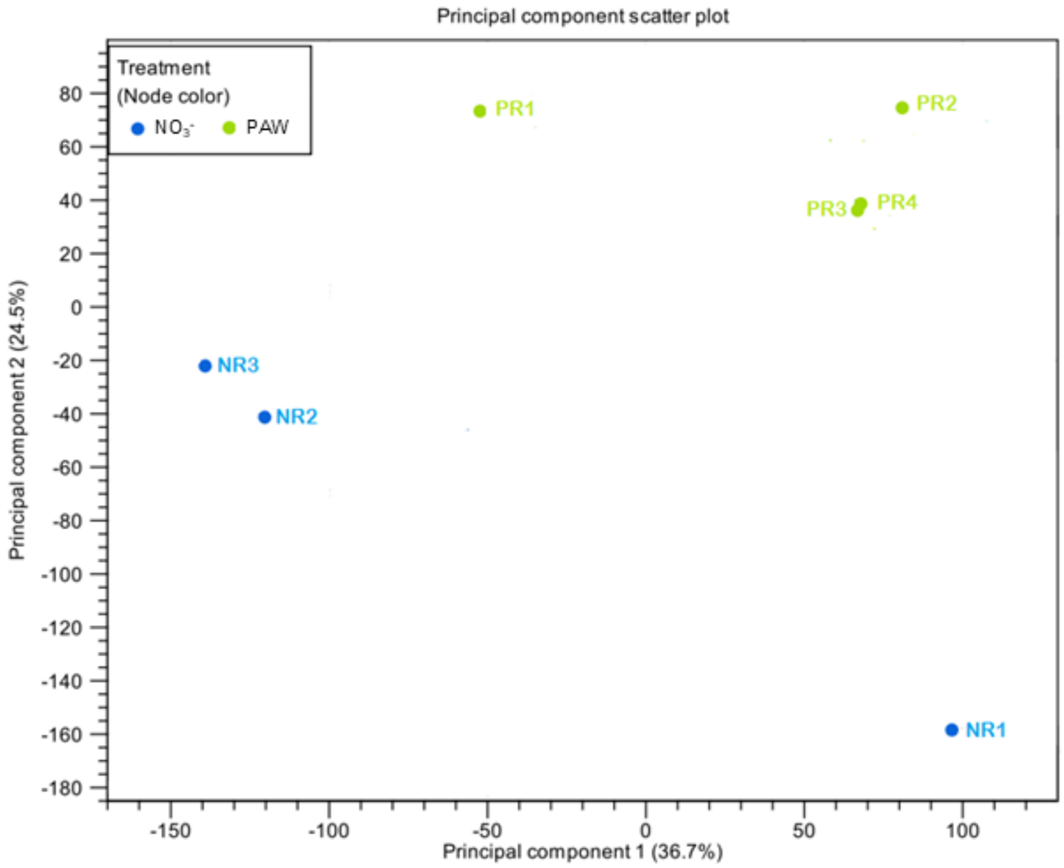

S3 Figure. Principal Component Analysis of root tissue samples.

Three-day old Arabidopsis seedlings were transferred to N-free media supplemented with PAW4 or 2.4mM NO_3^- as a control. Roots and shoots were harvested one week later, and RNA was extracted and sequenced. A principal component analysis (PCA) was conducted for the sequenced RNA. 4 PAW-treated root replicates (PR) and 3 NO_3^- control-treated root replicates were plotted.
